# Crystal structure of an iridium(III) complex of the [C(dppm)_2_] PCP pincer ligand system and its conjugate CH acid form

**DOI:** 10.1107/S2056989018004905

**Published:** 2018-04-06

**Authors:** Christian Reitsamer, Inge Schlapp-Hackl, Gabriel Partl, Walter Schuh, Holger Kopacka, Klaus Wurst, Paul Peringer

**Affiliations:** a University of Innsbruck, Faculty of Chemistry and Pharmacy, Innrain 80-82, 6020 Innsbruck, Austria

**Keywords:** iridium (III), PCP pincer, carbodi­phospho­rane (CDP), crystal structure, NMR

## Abstract

After the successful creation of the newly designed PCP carbodi­phospho­rane (CDP) ligand [Reitsamer *et al.* (2012 ▸). *Dalton Trans.* **41**, 3503–3514; Stallinger *et al.* (2007 ▸). *Chem. Commun.* pp. 510–512], the treatment of this PCP pincer system with the transition metal iridium and further the analysis of the structures by single-crystal diffraction and by NMR spectroscopy were of major inter­est. Two different iridium complexes, namely (bis­{[(di­phenyl­phosphan­yl)meth­yl]di­phenyl­phosphanyl­idene}methane-κ^3^ *P*,*C*,*P*′)carbonyl­chlorido­hydridoiridium(III) chloride di­chloro­methane tris­olvate, [Ir^III^(CO){C(dppm)_2_-κ^3^ *P*,*C*,*P*′}ClH]Cl·3CH_2_Cl_2_ (**1**) and the closely related (bis­{[(di­phenyl­phosphan­yl)meth­yl]di­phenyl­phosphanyl­idene}methanide(1+)-κ^3^ *P*,*C*,*P*′)carbonyl­chlorido­hy­dridoirid­ium(III) dichloride–hydro­chloric acid–water (1/2/5.5), [Ir^III^(CO){CH(dppm)_2_-κ^3^ *P*,*C*,*P*′)ClH]Cl}_2_ (**2**), have been designed and both complexes show a slightly distorted octa­hedral coordinated Ir^III^ centre. The PCP pincer ligand system is arranged in a *meridional* manner, the CO ligand is located *trans* to the central PCP carbon and a hydride and chloride are located perpendicular above and below the P_2_C_2_ plane. With an Ir—C_CDP_ distance of 2.157 (5) Å, an Ir—CO distance of 1.891 (6) Å and a quite short C—O distance of 1.117 (7) Å, complex **1** presents a strong carbonyl bond. Complex **2**, the corresponding CH acid of **1**, shows an additionally attached proton at the carbodi­phospho­rane carbon atom located *anti­periplanar* to the hydride of the metal centre. In comparison with complex **1**, the Ir—C_CDP_ distance of 2.207 (3) Å is lengthened and the Ir—C—O values indicate a weaker *trans* influence of the central carbodi­phospho­rane carbon atom.

## Abstract 2

The crystal structures of [Ir^III^(CO)(C(dppm)_2_-*κ* ^3^P,*C*,*P*′)ClH]Cl and [Ir^III^(CO)(CH(dppm)_2_-*κ* ^3^P,*C*,*P*′)ClH]Cl_2_ have been determined. Both complexes show a slightly distorted octa­hedral coordinated Ir^III^ centre. The PCP pincer ligand system is arranged in a *meridional* manner.

## Chemical context   

Based on a great number of investigations of iridium complexes in organic synthesis (Oro & Claver, 2009 ▸), on the large variety of metal–pincer ligand inter­actions and reactivities (Morales-Morales & Jensen, 2007 ▸; Choi *et al.*, 2011 ▸), the catalytic and stoichiometric organometallic chemistry of iridium PCP pincer complexes attracted our attention.

Up to now, diverse PCP pincer systems have been generated and these systems are, in general, classified according to the charge of the central carbon atom. Both an anionic *sp^2^* or *sp^3^* hybridization of the central carbon atom is possible (Table 1 ▸) and the charge arises from the metallation of the pertinent C—H functionalities of the non-coordinated ligand subunits. Furthermore, neutral PCP pincer ligands containing a divalent carbon(II) donor atom, for instance an alkyl­idene carbene or a NHC, are well known (Table 1 ▸; Crocker *et al.*, 1982 ▸). Moreover, PCP pincer complexes based on tropylium backbones have been reported. The cationic central carbon atom is part of a seven-membered six-electron arene fragment and because of the C—C bond lengths, designation as a cyclo­hepta­trienyl­idene carbene is allowed (Table 1 ▸).

Our focus is on the creation of new iridium complexes containing a PCP ligand system with a neutral or a cationic central carbon atom, respectively. The central carbon is part of a carbodi­phospho­rane (CDP) functionality and can be described as a naked carbon atom or as a divalent carbon(0) atom in an excited singlet (1*D*) state stabilized by two tertiary phosphines *via* donor–acceptor inter­actions. Consequently, this central atom disposes of two lone-electron pairs and is able to inter­act with one or two Lewis acids (Petz & Frenking, 2010 ▸).
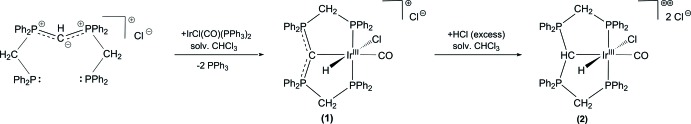


The protonated CDP ligand system [CH(dppm)_2_]Cl enters an oxidative addition reaction with Vaska’s compound [Ir^I^(CO)Cl(PPh_3_)_2_], forming the iridium PCP pincer CDP complex [Ir^III^(CO)(C(dppm)_2_-*κ* ^3^P,C,P′)ClH]Cl (**1**) (see reaction scheme). During this reaction sequence, the central carbon atom is deprotonated, becomes neutral and coordin­ates the iridium transition metal. Treatment of complex **1** with hydro­chloric acid leads to the protonation of the central carbon atom and consequently to the formation of the conjugate CH acid of **1**, the [Ir(CO)(CH(dppm)_2_-*κ* ^3^P,C,P′)ClH]Cl_2_ complex **2** (see reaction scheme[Chem scheme1]). Relative to the hydrido ligand at the iridium transition metal, the additionally attached proton adopts a *syn-* or *anti-periplanar* conformation. In solution, the existence of both isomers can be demonstrated by the use of NMR spectroscopy. However, the examination of several crystals revealed only the *anti-periplanar* configuration of complex **2**. Whether this is incidental or the crystallization is accompanied by the isomerization of the *syn-periplanar* to the *anti-periplanar* conformation is unclear.

## Structural commentary   

**Figure 1:**
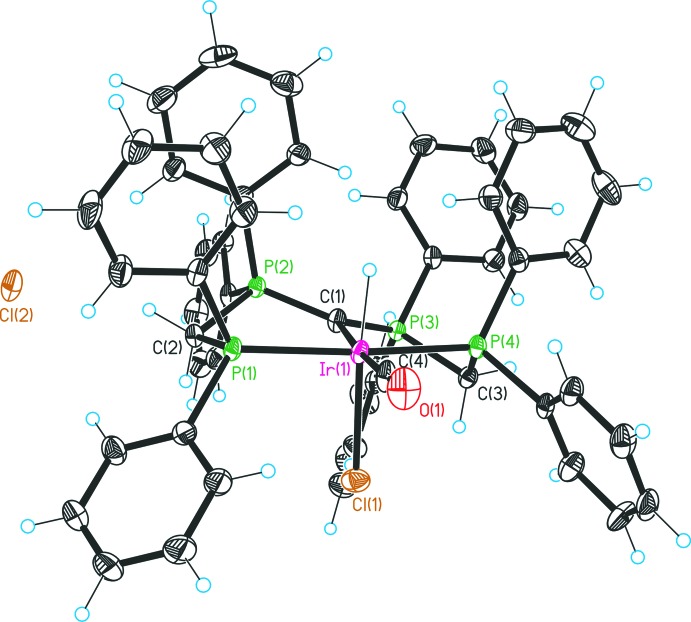
Structure of complex **1** with displacement ellipsoids drawn at the 30% probability level. Solvent residues are omitted.

Complex **1** (Fig. 1 ▸) crystallizes in the monoclinic space group *P*2_1_/*n* and the asymmetric unit consists of one formula unit of **1** and three mol­ecules of CH_2_Cl_2_. The structure can be divided into two parts, the [Ir^III^(CO)(C(dppm)_2_-*κ* ^3^P,C,P′)ClH]^+^ monocation and the chloride counter-ion. The iridium transition metal centre exhibits an octa­hedral ligand system, formed by a *meridional* arranged C(dppm)_2_, relative to the C1 atom, a *trans-*coordinated carbonyl unit, and a chlorido and hydrido ligand located perpendicular to the *meridional* plane or more precisely *trans* to each other. The P1—Ir1—P4 angle of 170.69 (5)° indicates a small deviation from the octa­hedral geometry and this value is larger compared to many related Iridium PCP pincer complexes. The environment of the CDP carbon atom C1 is strictly planar (sum of angles at C1 = 360°; Table 2 ▸) and the C1—P2 and C1—P3 bond lengths are 1.697 (5) and 1.711 (5) Å, respectively. Not only the geometry, but also the bond lengths are characteristic for a carbodi­phospho­rane atom, which inter­acts with one Lewis acid (Petz & Frenking, 2010 ▸). In general, bond lengths are directly connected with the valence-bond structure of a carbon atom and an increasing of the valence state causes a significant expansion of the bond gaps [C*sp* ^2^ < C(carbene) < C*sp* ^3^]. Consequently, the Ir1—C1 separation of 2.157 (5) Å indicates an *sp* ^3^ hybridization of the carbodiphosphorane carbon atom, which is substantiated by the data collected in Table 1 ▸. Additionally, inter­actions (Table 3 ▸) between the chloride counter-ion and the methyl­ene groups of the PCP pincer ligand system can be detected and the bond lengths of about 2.60 Å [Cl2⋯H2*B*(1 + *x*, *y*, *z*)] and 2.62 Å [Cl2⋯H3*B*(1 + *x*, *y*, *z*)] illustrate the location within the van der Waals radii. These C—H⋯*X* inter­actions are a common feature of complexes containing dppm or related ligands (Jones & Ahrens, 1998 ▸). Moreover, the chloride counter-ion inter­acts with the hydrogen atoms of the CH_2_Cl_2_ mol­ecules as well, forming distances of about 2.59 Å [Cl2⋯H5*B*(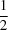 + *x*, 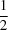 − *y*, 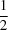 + *z*)] and 2.47 Å [Cl2⋯H6*B*(−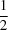 − *x*, 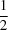 + *y*, 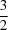 − *z*)].

**Figure 2:**
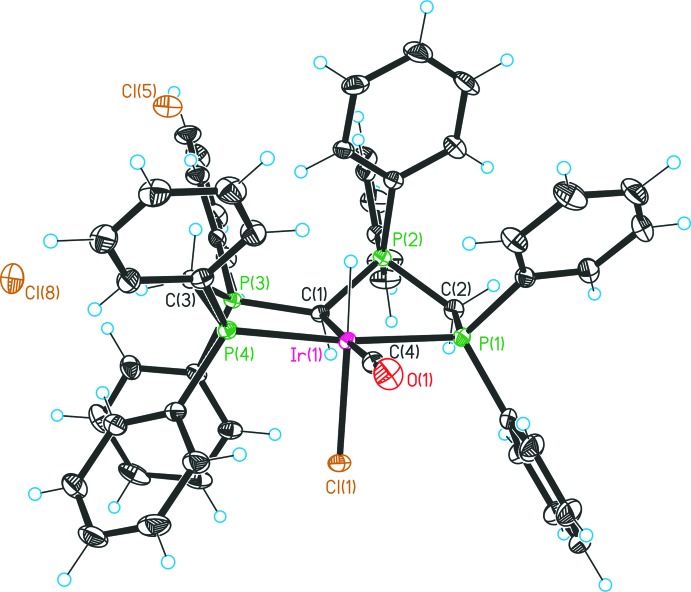
Structure of one of the two independent molecules of complex **2** with displacement ellipsoids drawn at the 30% probability level. Solvent residues are omitted.

The asymmetric unit of **2** comprises two [Ir^III^(CO)(CH(dppm)_2_-*κ* ^3^P,C,P′)ClH]Cl_2_ complex mol­ecules (Fig. 2 ▸), four mol­ecules of HCl and eleven mol­ecules of water in total. Both complex mol­ecules are distinctly asymmetric in the solid state. As a result of the threefold coordination of the transition metal by the PCP pincer ligand system, two five-membered metallacycles are formed, each adopting an approximately envelope conformation. One methyl­ene group (C3) and one phospho­rus atom (P2) are positioned at the flap positions above the plane generated by the C1–C2–P1–Ir1 and C1–Ir1–P3–P4 atoms. Complex **2** crystallizes in the monoclinic space group *P*2_1_/*n* and the complex mol­ecule can be described as one [Ir^III^(CO)(CH(dppm)_2_-*κ* ^3^P,C,P′)ClH]^2+^ dication stabilized by two chloride counter-ions. Overall, complex **2** represents the conjugate CH acid of the [Ir^III^(CO)(C(dppm)_2_-*κ* ^3^P,C,P′)ClH]Cl complex (**1**). The carbodi­phospho­rane carbon atom additionally coordinates a second Lewis acid, the proton H1, which adopts an *anti-periplanar* conformation relative to the hydrido ligand H11. As a consequence, atom C1 forms a distorted tetra­hedron with the directly coordinated atoms (sum of angles = 344.3°). In comparison with complex **1**, the values of the angles P2—C1–Ir1 and P3—C1—Ir1 are significantly reduced, whereas the P2—C1—P3 angles differs to a lesser extent (Table 2 ▸). The coordination of a second Lewis acid causes a lengthening of the C1—P distances by about 0.098 Å, resulting in bond lengths in the range of P—C single bonds. Moreover, the Ir1—C1 distance [2.207 (3) Å] is markedly longer compared to that of the conjugate base **1** [2.157 (5) Å], as has also been observed in other carbodi­phospho­rane complexes (Petz *et al.*, 2009 ▸; Reitsamer *et al.*, 2012 ▸; Tonner *et al.*, 2006 ▸). Furthermore, the protonation of the C1 atom leads to a decrease of the *trans* influence of the carbodi­phospho­rane carbon donor atom, confirmed by an shortening of the Ir—CO distance and an increasing of the carbonyl bond gap. Besides, C—H⋯O and C—H⋯Cl interactions (Table 4 ▸) between the methylene groups of the dppm moieties and the water or HCl molecules can be detected, causing for example separations in the range of 2.61 Å [H2*A*⋯O4(1 − *x*, 1 − *y*, 1 − *z*)], 2.89 Å [H2*B*⋯Cl7(*x* − 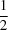, −*y* + 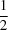, *z* + 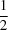)], 2.51 Å [H3*A*⋯Cl8(*x*, 1 + *y*, *z*)] and 2.57 Å [H3*B*⋯Cl5(*x*, 1 + *y*, *z*)].

In **1**, there are inter­actions (Table 3 ▸) between the chloride counter-ion and the methyl­ene groups of the PCP pincer ligand system [Cl2⋯H2*B* = 2.60 Å, H3*B*⋯Cl2(1 + *x*, *y*, *z*) = 2.62 Å] with distances shorter than the sum of the van der Waals radii. Such C—H⋯*X* inter­actions are a common feature of complexes containing dppm or related ligands (Jones & Ahrens, 1998 ▸). Moreover, the chloride counter-ion also inter­acts with the hydrogen atoms of the CH_2_Cl_2_ mol­ecules [H5*B*⋯Cl2(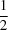 + *x*, 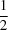 − *y*, 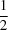 + *z*) = 2.59 Å and H5*B*⋯Cl2(−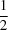 − *x*, 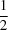 + *y*, 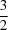 − *z*) = 2.47 Å].

In **2**, C—H⋯O and C—H⋯Cl inter­actions (Table 4 ▸) occur between the methyl­ene groups of the dppm moieties and the water or HCl mol­ecules and there are short contacts of 2.61 Å [H2*A*⋯O4(1 − *x*, 1 − *y*, 1 − *z*)], 2.89 Å [H2*B*⋯Cl7(*x* − 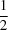, −*y* + 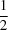, *z* + 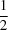)], 2.51 Å [H3*A*⋯Cl8(*x*, 1 + *y*, *z*)] and 2.57 Å [H3*B*⋯Cl5(*x*, 1 + *y*, *z*)]. A network of different inter­actions occurs between the two independent complex mol­ecules. The water and hydro­chloric acid solvent mol­ecules form hydrogen bonds with the chloride ligands or counter-ions and the hydrogen atoms of the complex mol­ecules, respectively.

## Synthesis and crystallization   

All preparations were carried out under an inert atmosphere (N_2_) using standard Schlenk techniques. The ^1^H, ^13^C and ^31^P NMR spectra were recorded on a Bruker DPX 300 NMR spectrometer and were referenced against the ^13^C/^1^H solvent peaks of the solvents chloro­form, methanol or the external 85% H_3_PO_4_ standard, respectively. The phospho­rus atoms in the NMR data are labelled as in Figs. 1 ▸ and 2 ▸.

**Synthesis of [Ir(CO)(C(dppm)_2_-** ***κ*** **^3^P,C,P′)ClH]Cl (1):** A mixture of 19.5 mg of Vaska’s complex (0.025 mmol), 20.4 mg of [CH(dppm)_2_]Cl (0.025 mmol) (Reitsamer *et al.*, 2012 ▸) and CHCl_3_ (0.6 ml) was stirred at ambient temperature for 15 min. The solvent was evaporated *in vacuo* and the residue was digested with a mixture of CH_2_Cl_2_ (0.1 ml) and ethyl acetate (0.7 ml). The solid was separated and washed twice with ethyl acetate (0.6 ml). Single crystals were grown by slow evaporation of a solution in CH_2_Cl_2_. ^31^P {^1^H} NMR (CHCl_3_): δ 31.9 (P2/P3, N = 71), δ 8.2 (P1/P4); ^13^C {^1^H} NMR (CDCl_3_): δ −4.4 (C1, ^1^ *J*_P2/P3C1_ = 86, ^1^ *J*_P1/P4C1_ = 6, ^1^ *J*_C1H(11)_ = 4); ^1^H NMR (CDCl_3_): δ −16.7 (H11, ^1^ *J*_P1/P4H11_ = 10).

**Synthesis of [Ir(CO)(CH(dppm)_2_-** ***κ*** **^3^P,C,P′)ClH]Cl_2_ (2):** 19.5 mg of Vaska’s complex (0.025 mmol) and 20.4 mg of [CH(dppm)_2_]Cl (0.025 mmol) (Reitsamer *et al.*, 2012 ▸) were solved in CHCl_3_ (0.6 ml). The mixture was stirred at ambient temperature for 15 min. After addition of 0.1 ml of hydro­chloric acid (10 mol L^−1^), the product crystallized upon standing for a day. ^31^P {^1^H} NMR (CHCl_3_/MeOH): δ 45.3 (P2/P3, N = 61), δ 1.7 (P1/P4); ^13^C {^1^H} NMR (CDCl_3_): δ 9.1 (C1, ^1^ *J*_P2/P3C1_) = 38, ^1^ *J*_C1H1_ = 122); ^1^H NMR (CDCl_3_/MeOH): δ −18.9 (H11, ^1^ *J*_P1/P4H11_ = 11).

## Refinement   

Crystal data, data collection and structure refinement details are summarized in Table 5 ▸. Refinement of complex **1** resulted in the location of the hydride hydrogen atom. The bond length was restrained to a distance of 1.6 Å and a fixed isotropic displacement parameter of 1.5*U*_eq_ of iridium was applied. The hydrido ligand of complex **2** was also detected and refined isotropically without the use of bond restraints. Furthermore, the proton of the CDP carbon atom was spotted and refined with bond restraints of 0.98 Å. The hydrogen atoms of the water and solvent mol­ecules could only be partially detected and were omitted. A determination of a 1:1 positional disorder of one water mol­ecule (O4 and O4*A*) and one HCl or chloride (Cl10 and Cl1*A*) was possible. Eight chloride positions can be detected, which are occupied by a total of four chlorides and four hydro­chloric acid units. The hydrogen-atom positions of the phenyl subunits and methyl­ene groups were refined with calculated positions (C—H = 0.94 and 0.98 Å) using a riding model with *U*_iso_(H) = 1.2*U*_eq_(C).

The two complex mol­ecules of **2** are related to each other by the presence of a pseudo-symmetry centre. A halving of the *c* axis and consequently the changing of the monoclinic setting from *P*2_1_/*n* to *P*2_1_/*c* allows the consideration of one formula unit of **2**. A closer observation of the sections of the reciprocal lattice along c* (*l* = 2*n* + 1) at different values of *l* results in the presence of frequent weak reflections. Consequently, an inter­pretation of this system as three-dimensional network between two complex mol­ecules, four hydro­chloric acid units and eleven water mol­ecules allows the involvement of these weak, but clearly existing reflections, and establishes the possibility of the distinction of the chloride and oxygen positions.

## Supplementary Material

Crystal structure: contains datablock(s) global, complex1, complex2. DOI: 10.1107/S2056989018004905/eb2007sup1.cif

Structure factors: contains datablock(s) complex1. DOI: 10.1107/S2056989018004905/eb2007complex1sup2.hkl

Structure factors: contains datablock(s) complex2. DOI: 10.1107/S2056989018004905/eb2007complex2sup3.hkl

CCDC references: 1577807, 1577808

Additional supporting information:  crystallographic information; 3D view; checkCIF report

## Figures and Tables

**Table 1:** Comparative Ir—C_PCP_ and Ir—C_CO_ bond lengths (Å) of different [Ir(CO)ClH(PCP)] complexes

PCP ligand or backbone (charges are omitted)	PCP central carbon atom	Ir—C_CO_	Ir—C_PCP_	Reference
C_6_H_3_-1,3-[OP*R*_2_]_2_	*sp* ^2^	2.045 (3)	1.949 (4)	Goldberg *et al.* (2015 ▸)
C_6_H_3_-1,3-[OP*R*_2_]_2_	*sp* ^2^	2.057 (3)	1.913 (4)	Goldberg *et al.* (2015 ▸)
C_6_H_3_-1,3-[OP*R*_2_]_2_	*sp* ^2^	2.071 (2)	1.921 (3)	Goldberg *et al.* (2015 ▸)
C(NCH_2_P*R*_2_)_2_C_10_H_6_	NHC	2.078 (4)	1.904 (5)	Hill & McQueen (2012 ▸)
benzotropylium	alkyl­idene	2.082	1.929	Leis *et al.* (2014 ▸)
tropylium	alkyl­idene	2.093 (5)	1.916 (5)	Winter *et al.* (2005 ▸)
C_6_H_3_-1,3-[P(CF_3_)_2_]_2_	*sp* ^2^	2.103 (2)	1.952 (3)	Adams *et al.* (2011 ▸)
C_3_H_3_-1,2-[OP*R*_2_]_2_	*sp* ^3^	2.126 (8)	1.880 (7)	Ruhland & Herdtweck (2005 ▸)
CH(NCH_2_P*R*_2_)_2_C_10_H_6_	*sp* ^3^	2.141 (5)	1.904 (6)	Hill & McQueen (2012 ▸)
C(dppm)_2_	CDP	2.157 (5)	1.891 (6)	this work
cyclo­hex­yl	*sp* ^3^	2.159 (4)	1.909 (5)	Jonasson *et al.* (2015 ▸)
trypticene	*sp* ^3^	2.163 (2)	1.895 (2)	Azerraf & Gelman (2009 ▸)
cyclo­hex­yl	*sp* ^3^	2.165 (5)	1.906 (6)	Mayer *et al.* (1993 ▸)
trypticene	*sp* ^3^	2.193(3	1.898 (3)	Azerraf & Gelman (2009 ▸)
CH(dppm)_2_	protonated CDP	2.207 (3)	1.874 (4)	this work
cyclo­hepta­trien­yl	*sp* ^3^	2.25 (2)	1.78 (1)	Nemeh *et al.* (1998 ▸)

**Table 2:** Selected distances and angles (Å, °) of **1** and **2**

	complex **1**	complex **2** ^*a*^
Ir1—C1	2.157 (5)	2.207 (3)
Ir1—C4	1.891 (6)	1.874 (4)
Ir1—P1	2.344 (1)	2.347 (1)
Ir1—P4	2.315 (2)	2.332 (1)
Ir1—H1	1.54 (3)	1.52 (4)
C4—O1	1.117 (7)	1.135 (5)
P1—C2	1.827 (5)	1.837 (4)
P2—C2	1.800 (5)	1.803 (4)
P2—C1	1.697 (5)	1.802 (3)
P3—C1	1.711 (5)	1.801 (3)
P2—C1—P3	125.7 (3)	122.1 (2)
P2—C1—Ir1	113.9 (3)	107.8 (2)
P3—C1—Ir1	120.4 (3)	114.5 (2)
P4—Ir1—P1	170.7 (1)	171.9 (1)

Note: (*a*) the second independent formula unit displays similar values.

**Table 3:** Hydrogen-bond geometry (Å, °) for complex **1** [Chem scheme1]

*D*—H⋯*A*	*D*—H	H⋯*A*	*D*⋯*A*	*D*—H⋯*A*
C2—H2*B*⋯Cl2	0.98	2.60	3.534 (5)	160
C3—H3*B*⋯Cl2^i^	0.98	2.62	3.544 (5)	157
C5—H5*B*⋯Cl2^ii^	0.98	2.59	3.541 (10)	165
C6—H6*B*⋯Cl2^iii^	0.98	2.47	3.436 (10)	169
C7—H7*A*⋯Cl1^iv^	0.98	2.50	3.447 (15)	163
C105—H105⋯Cl1^v^	0.94	2.72	3.573 (8)	151
C312—H312⋯Cl2^i^	0.94	2.78	3.690 (6)	163

Symmetry codes: (i) 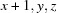; (ii) 
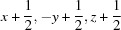
; (iii) 
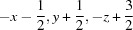
; (iv) 
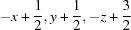
; (v) 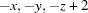.

**Table 4:** Hydrogen-bond geometry (Å, °) for complex **2** [Chem scheme1]

*D*—H⋯*A*	*D*—H	H⋯*A*	*D*⋯*A*	*D*—H⋯*A*
C1—H1⋯Cl1	0.96 (3)	2.82 (3)	3.252 (3)	109 (2)
C3—H3*A*⋯Cl8^i^	0.98	2.51	3.466 (4)	164
C3—H3*B*⋯Cl5^i^	0.98	2.57	3.493 (4)	158
C6—H6*A*⋯O8^ii^	0.98	2.59	3.431 (5)	144
C6—H6*B*⋯Cl9^ii^	0.98	2.82	3.746 (4)	158
C7—H7*A*⋯Cl1*A* ^iii^	0.98	2.73	3.614 (6)	150
C7—H7*B*⋯Cl4^iii^	0.98	2.60	3.518 (4)	157
C206—H206⋯Cl7^iii^	0.94	2.79	3.719 (4)	172
C310—H310⋯Cl4^ii^	0.94	2.83	3.714 (4)	158
C602—H602⋯Cl9^ii^	0.94	2.62	3.557 (4)	179
C704—H704⋯Cl1^iv^	0.94	2.82	3.534 (6)	134
C708—H708⋯Cl2	0.94	2.80	3.503 (4)	132
C710—H710⋯Cl5^v^	0.94	2.72	3.614 (4)	160
C712—H712⋯Cl10^iii^	0.94	2.81	3.734 (6)	167

Symmetry codes: (i) 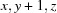; (ii) 
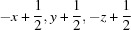
; (iii) 
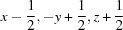
; (iv) 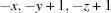; (v) 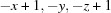.

**Table 5:** Experimental details

	complex **1**	complex **2**
Crystal data		
Chemical formula	[IrClH(CO)(C_51_H_44_P_4_)]Cl·3CH_2_Cl_2_	[IrClH(C_51_H_44_P_4_)(CO)]Cl_2_·2HCl·5.5H_2_O
*M*_r_	1327.64	1281.32
Crystal system, space group	Monoclinic, *P*2_1_/*n*	Monoclinic, *P*2_1_/*n*
Temperature (K)	233	233
*a*, *b*, *c* (Å)	12.3477 (2), 24.7472 (5), 19.0123 (3)	19.7138 (2), 22.7327 (2), 25.3120 (3)
β (°)	91.700 (1)	98.781 (1)
*V* (Å^3^)	5807.05 (18)	11210.6 (2)
*Z*	4	8
Radiation type	Mo *K*α	Mo *K*α
μ (mm^−1^)	2.82	2.78
Crystal size (mm)	0.3 × 0.15 × 0.05	0.3 × 0.2 × 0.06

Data collection		
Diffractometer	Nonius KappaCCD	Nonius KappaCCD
No. of measured, independent and observed [*I* > 2σ(*I*)] reflections	33007, 10203, 8088	68943, 22078, 17290
*R*_int_	0.061	0.037

Refinement		
*R*[*F* ^2^ > 2σ(*F* ^2^)], *wR*(*F* ^2^), *S*	0.043, 0.098, 1.06	0.033, 0.088, 1.04
No. of reflections	10203	22078
No. of parameters	625	1268
No. of restraints	1	2
H-atom treatment	H-atom parameters constrained	H-atom parameters constrained
		
Δρ_max_, Δρ_min_ (e Å^−3^)	1.68, −0.91	2.03, −0.99

Computer programs: *COLLECT* Nonius, 1999 ▸, *DENZO* and *SCALEPACK* (Otwinowski & Minor, 1997 ▸), *SHELXS97* and *SHELXL97* (Sheldrick, 2008 ▸) and *ChemDraw* (Cambridge Soft, 2001 ▸).

## supplementary crystallographic information

### (Bis{[(diphenylphosphanyl)methyl]diphenylphosphanylidene}methane-\ κ3P,C,P')carbonylchloridohydridoiridium(III) chloride dichloromethane trisolvate (complex1) Crystal data

**Table:**

[IrClH(CO)(C_51_H_44_P_4_)]Cl·3CH_2_Cl_2_	*F*(000) = 2648
*M**_r_* = 1327.64	*D*_x_ = 1.519 Mg m^−^^3^
Monoclinic, *P*2_1_/*n*	Mo *K*α radiation, λ = 0.71073 Å
*a* = 12.3477 (2) Å	Cell parameters from 76576 reflections
*b* = 24.7472 (5) Å	θ = 1.0–25.0°
*c* = 19.0123 (3) Å	µ = 2.82 mm^−^^1^
β = 91.700 (1)°	*T* = 233 K
*V* = 5807.05 (18) Å^3^	Prism, colorless
*Z* = 4	0.3 × 0.15 × 0.05 mm

### (Bis{[(diphenylphosphanyl)methyl]diphenylphosphanylidene}methane-\ κ3P,C,P')carbonylchloridohydridoiridium(III) chloride dichloromethane trisolvate (complex1) Data collection

**Table:**

Nonius KappaCCD diffractometer	8088 reflections with *I* > 2σ(*I*)
Radiation source: fine-focus sealed tube	*R*_int_ = 0.061
Graphite monochromator	θ_max_ = 25.0°, θ_min_ = 1.4°
phi– and ω–scans	*h* = −14→13
33007 measured reflections	*k* = −29→29
10203 independent reflections	*l* = −22→22

### (Bis{[(diphenylphosphanyl)methyl]diphenylphosphanylidene}methane-\ κ3P,C,P')carbonylchloridohydridoiridium(III) chloride dichloromethane trisolvate (complex1) Refinement

**Table:**

Refinement on *F*^2^	Primary atom site location: structure-invariant direct methods
Least-squares matrix: full	Secondary atom site location: difference Fourier map
*R*[*F*^2^ > 2σ(*F*^2^)] = 0.043	Hydrogen site location: inferred from neighbouring sites
*wR*(*F*^2^) = 0.098	H-atom parameters constrained
*S* = 1.06	*w* = 1/[σ^2^(*F*_o_^2^) + (0.0396*P*)^2^ + 13.9388*P*] where *P* = (*F*_o_^2^ + 2*F*_c_^2^)/3
10203 reflections	(Δ/σ)_max_ = 0.002
625 parameters	Δρ_max_ = 1.68 e Å^−^^3^
1 restraint	Δρ_min_ = −0.91 e Å^−^^3^

### (Bis{[(diphenylphosphanyl)methyl]diphenylphosphanylidene}methane-\ κ3P,C,P')carbonylchloridohydridoiridium(III) chloride dichloromethane trisolvate (complex1) Special details

**Table:**

Experimental. All data sets were measured with several scans to increase the number of redundant reflections. In our experience this method of averaging redundant reflections replaces in a good approximation semi-empirical absorptions methods (absorption correction programs like SORTAV lead to no better data sets).
Geometry. All esds (except the esd in the dihedral angle between two l.s. planes) are estimated using the full covariance matrix. The cell esds are taken into account individually in the estimation of esds in distances, angles and torsion angles; correlations between esds in cell parameters are only used when they are defined by crystal symmetry. An approximate (isotropic) treatment of cell esds is used for estimating esds involving l.s. planes.
Refinement. The hydrogen atom at Ir1 were found and must be refined with bond restraint of 1.6 angs. and a fixed isotropc displacement parameter of 1.5 times higher than Ueq of Ir1.

### (Bis{[(diphenylphosphanyl)methyl]diphenylphosphanylidene}methane-\ κ3P,C,P')carbonylchloridohydridoiridium(III) chloride dichloromethane trisolvate (complex1) Fractional atomic coordinates and isotropic or equivalent isotropic displacement parameters (Å^2^)

**Table:**

	*x*	*y*	*z*	*U*_iso_*/*U*_eq_	
Ir1	0.086262 (15)	0.157429 (8)	0.860593 (10)	0.02760 (8)	
H1	0.071 (4)	0.2193 (11)	0.863 (3)	0.041*	
Cl1	0.12882 (13)	0.05923 (6)	0.85269 (8)	0.0467 (4)	
Cl2	−0.41779 (12)	0.14124 (7)	0.72730 (9)	0.0543 (4)	
P1	−0.10105 (10)	0.14212 (5)	0.85315 (7)	0.0291 (3)	
P2	−0.04100 (10)	0.17204 (5)	0.71034 (7)	0.0264 (3)	
P3	0.20212 (10)	0.15691 (5)	0.70266 (6)	0.0258 (3)	
P4	0.26873 (10)	0.17562 (6)	0.84860 (7)	0.0285 (3)	
O1	0.0951 (4)	0.1610 (2)	1.0188 (2)	0.0661 (14)	
C1	0.0843 (4)	0.1623 (2)	0.7473 (3)	0.0295 (11)	
C2	−0.1350 (4)	0.1327 (2)	0.7597 (3)	0.0287 (11)	
H2A	−0.1294	0.0945	0.7472	0.034*	
H2B	−0.2093	0.1448	0.7493	0.034*	
C3	0.3047 (4)	0.1398 (2)	0.7698 (3)	0.0305 (11)	
H3A	0.3056	0.1008	0.7783	0.037*	
H3B	0.3766	0.1510	0.7549	0.037*	
C4	0.0912 (4)	0.1583 (2)	0.9601 (3)	0.0410 (14)	
C101	−0.1556 (4)	0.0815 (2)	0.8934 (3)	0.0378 (13)	
C102	−0.2431 (5)	0.0545 (3)	0.8631 (3)	0.0489 (15)	
H102	−0.2743	0.0671	0.8205	0.059*	
C103	−0.2854 (6)	0.0090 (3)	0.8950 (4)	0.0621 (19)	
H103	−0.3455	−0.0089	0.8743	0.074*	
C104	−0.2387 (6)	−0.0097 (3)	0.9572 (4)	0.067 (2)	
H104	−0.2655	−0.0412	0.9783	0.080*	
C105	−0.1535 (6)	0.0173 (3)	0.9883 (4)	0.067 (2)	
H105	−0.1236	0.0053	1.0315	0.080*	
C106	−0.1112 (5)	0.0624 (3)	0.9563 (3)	0.0519 (16)	
H106	−0.0516	0.0803	0.9775	0.062*	
C107	−0.1853 (4)	0.1971 (2)	0.8843 (3)	0.0328 (12)	
C108	−0.1433 (5)	0.2475 (3)	0.9003 (4)	0.0513 (16)	
H108	−0.0686	0.2536	0.8962	0.062*	
C109	−0.2081 (6)	0.2891 (3)	0.9222 (4)	0.0607 (19)	
H109	−0.1780	0.3234	0.9308	0.073*	
C110	−0.3162 (5)	0.2806 (3)	0.9313 (4)	0.0602 (19)	
H110	−0.3604	0.3088	0.9470	0.072*	
C111	−0.3597 (5)	0.2303 (3)	0.9173 (4)	0.064 (2)	
H111	−0.4338	0.2240	0.9242	0.076*	
C112	−0.2951 (5)	0.1891 (3)	0.8933 (4)	0.0522 (16)	
H112	−0.3260	0.1552	0.8829	0.063*	
C201	−0.0556 (4)	0.1498 (2)	0.6200 (3)	0.0307 (12)	
C202	−0.0488 (5)	0.0946 (2)	0.6065 (3)	0.0393 (13)	
H202	−0.0412	0.0700	0.6440	0.047*	
C203	−0.0531 (5)	0.0761 (3)	0.5384 (3)	0.0526 (16)	
H203	−0.0485	0.0388	0.5297	0.063*	
C204	−0.0640 (5)	0.1112 (3)	0.4826 (3)	0.0527 (17)	
H204	−0.0658	0.0979	0.4362	0.063*	
C205	−0.0723 (5)	0.1654 (3)	0.4949 (3)	0.0509 (16)	
H205	−0.0810	0.1895	0.4568	0.061*	
C206	−0.0677 (5)	0.1852 (2)	0.5635 (3)	0.0372 (13)	
H206	−0.0729	0.2225	0.5716	0.045*	
C207	−0.0969 (4)	0.2400 (2)	0.7139 (3)	0.0320 (12)	
C208	−0.0311 (5)	0.2827 (2)	0.7380 (3)	0.0420 (14)	
H208	0.0427	0.2769	0.7491	0.050*	
C209	−0.0760 (7)	0.3337 (3)	0.7453 (4)	0.0604 (19)	
H209	−0.0331	0.3625	0.7627	0.072*	
C210	−0.1832 (7)	0.3423 (3)	0.7272 (4)	0.069 (2)	
H210	−0.2127	0.3771	0.7308	0.083*	
C211	−0.2478 (6)	0.3003 (3)	0.7038 (4)	0.0639 (19)	
H211	−0.3211	0.3066	0.6918	0.077*	
C212	−0.2055 (5)	0.2493 (2)	0.6978 (3)	0.0429 (14)	
H212	−0.2503	0.2206	0.6828	0.051*	
C301	0.2117 (4)	0.1022 (2)	0.6384 (3)	0.0317 (12)	
C302	0.1958 (5)	0.0497 (2)	0.6616 (3)	0.0480 (15)	
H302	0.1760	0.0431	0.7082	0.058*	
C303	0.2094 (6)	0.0069 (3)	0.6153 (4)	0.064 (2)	
H303	0.1995	−0.0288	0.6306	0.077*	
C304	0.2373 (6)	0.0170 (3)	0.5470 (4)	0.070 (2)	
H304	0.2479	−0.0120	0.5160	0.084*	
C305	0.2497 (6)	0.0689 (3)	0.5237 (4)	0.066 (2)	
H305	0.2662	0.0756	0.4766	0.079*	
C306	0.2378 (6)	0.1115 (3)	0.5701 (3)	0.0518 (16)	
H306	0.2478	0.1472	0.5545	0.062*	
C307	0.2528 (4)	0.2156 (2)	0.6562 (2)	0.0294 (11)	
C308	0.1817 (5)	0.2551 (2)	0.6310 (3)	0.0448 (15)	
H308	0.1072	0.2515	0.6385	0.054*	
C309	0.2191 (5)	0.2996 (3)	0.5952 (4)	0.0550 (17)	
H309	0.1702	0.3258	0.5777	0.066*	
C310	0.3276 (6)	0.3051 (3)	0.5853 (3)	0.0532 (17)	
H310	0.3537	0.3358	0.5620	0.064*	
C311	0.3980 (5)	0.2664 (3)	0.6090 (3)	0.0498 (16)	
H311	0.4724	0.2705	0.6014	0.060*	
C312	0.3620 (5)	0.2216 (2)	0.6438 (3)	0.0423 (14)	
H312	0.4116	0.1950	0.6592	0.051*	
C401	0.3590 (4)	0.1508 (2)	0.9181 (3)	0.0320 (12)	
C402	0.3547 (5)	0.1748 (2)	0.9852 (3)	0.0431 (14)	
H402	0.3100	0.2051	0.9917	0.052*	
C403	0.4148 (5)	0.1547 (3)	1.0413 (3)	0.0528 (17)	
H403	0.4107	0.1709	1.0858	0.063*	
C404	0.4810 (5)	0.1109 (3)	1.0320 (3)	0.0548 (18)	
H404	0.5217	0.0971	1.0705	0.066*	
C405	0.4882 (6)	0.0872 (3)	0.9672 (4)	0.0612 (19)	
H405	0.5341	0.0574	0.9613	0.073*	
C406	0.4272 (5)	0.1073 (3)	0.9099 (3)	0.0488 (15)	
H406	0.4328	0.0911	0.8654	0.059*	
C407	0.3075 (4)	0.2449 (2)	0.8343 (3)	0.0332 (12)	
C408	0.2362 (5)	0.2876 (2)	0.8433 (3)	0.0436 (14)	
H408	0.1670	0.2807	0.8607	0.052*	
C409	0.2651 (7)	0.3400 (3)	0.8272 (4)	0.0613 (19)	
H409	0.2156	0.3684	0.8331	0.074*	
C410	0.3658 (7)	0.3504 (3)	0.8027 (4)	0.067 (2)	
H410	0.3851	0.3859	0.7908	0.080*	
C411	0.4388 (6)	0.3093 (3)	0.7955 (4)	0.066 (2)	
H411	0.5089	0.3170	0.7802	0.079*	
C412	0.4104 (5)	0.2564 (2)	0.8104 (3)	0.0483 (15)	
H412	0.4605	0.2283	0.8043	0.058*	
C5	0.1302 (10)	0.3639 (5)	1.0447 (5)	0.115 (4)	
H5A	0.2050	0.3771	1.0441	0.138*	
H5B	0.1056	0.3673	1.0930	0.138*	
Cl3	0.1294 (3)	0.29711 (13)	1.02240 (17)	0.1278 (10)	
Cl4	0.0511 (4)	0.4043 (2)	0.9911 (3)	0.211 (2)	
C6	−0.0035 (10)	0.5119 (4)	0.8155 (7)	0.125 (4)	
H6A	0.0440	0.5090	0.8576	0.150*	
H6B	−0.0163	0.5503	0.8062	0.150*	
Cl5	0.0611 (3)	0.48306 (14)	0.7439 (2)	0.1557 (14)	
Cl6	−0.1255 (4)	0.48058 (19)	0.8312 (3)	0.204 (2)	
C7	0.5564 (14)	0.4630 (5)	0.7031 (10)	0.171 (6)	
H7A	0.4951	0.4878	0.6961	0.205*	
H7B	0.6196	0.4799	0.6822	0.205*	
Cl7	0.5296 (4)	0.40838 (13)	0.66066 (17)	0.1600 (16)	
Cl8	0.5822 (3)	0.45621 (16)	0.7924 (3)	0.1825 (19)	

### (Bis{[(diphenylphosphanyl)methyl]diphenylphosphanylidene}methane-\ κ3P,C,P')carbonylchloridohydridoiridium(III) chloride dichloromethane trisolvate (complex1) Atomic displacement parameters (Å^2^)

**Table:**

	*U*^11^	*U*^22^	*U*^33^	*U*^12^	*U*^13^	*U*^23^
Ir1	0.02108 (11)	0.03984 (13)	0.02192 (11)	−0.00021 (9)	0.00161 (7)	0.00209 (9)
Cl1	0.0525 (9)	0.0430 (8)	0.0451 (8)	0.0033 (7)	0.0104 (7)	0.0113 (6)
Cl2	0.0278 (7)	0.0736 (11)	0.0614 (10)	0.0034 (7)	−0.0001 (7)	0.0024 (8)
P1	0.0210 (6)	0.0401 (8)	0.0262 (7)	−0.0005 (6)	0.0029 (5)	0.0042 (6)
P2	0.0223 (6)	0.0338 (7)	0.0230 (7)	0.0004 (5)	0.0009 (5)	0.0016 (5)
P3	0.0228 (6)	0.0337 (7)	0.0209 (6)	−0.0002 (5)	0.0016 (5)	0.0012 (5)
P4	0.0210 (6)	0.0400 (7)	0.0247 (7)	−0.0015 (6)	0.0021 (5)	0.0004 (6)
O1	0.068 (3)	0.104 (4)	0.027 (3)	−0.009 (3)	0.004 (2)	−0.004 (2)
C1	0.024 (3)	0.044 (3)	0.021 (2)	−0.002 (2)	0.004 (2)	0.003 (2)
C2	0.024 (3)	0.037 (3)	0.025 (3)	−0.005 (2)	0.001 (2)	0.000 (2)
C3	0.026 (3)	0.038 (3)	0.028 (3)	0.002 (2)	0.002 (2)	0.002 (2)
C4	0.022 (3)	0.064 (4)	0.037 (4)	−0.003 (3)	0.002 (2)	−0.001 (3)
C101	0.033 (3)	0.042 (3)	0.039 (3)	0.003 (3)	0.012 (3)	0.010 (3)
C102	0.044 (4)	0.056 (4)	0.047 (4)	−0.012 (3)	0.005 (3)	0.009 (3)
C103	0.047 (4)	0.056 (4)	0.084 (5)	−0.016 (3)	0.006 (4)	0.009 (4)
C104	0.056 (5)	0.058 (4)	0.087 (6)	−0.011 (4)	0.019 (4)	0.028 (4)
C105	0.062 (5)	0.071 (5)	0.068 (5)	0.002 (4)	0.008 (4)	0.037 (4)
C106	0.045 (4)	0.055 (4)	0.055 (4)	0.000 (3)	0.004 (3)	0.019 (3)
C107	0.025 (3)	0.049 (3)	0.024 (3)	0.001 (2)	0.001 (2)	−0.001 (2)
C108	0.040 (4)	0.056 (4)	0.058 (4)	0.003 (3)	0.018 (3)	−0.011 (3)
C109	0.053 (4)	0.055 (4)	0.076 (5)	0.001 (3)	0.022 (4)	−0.020 (4)
C110	0.046 (4)	0.072 (5)	0.063 (4)	0.019 (4)	0.012 (3)	−0.021 (4)
C111	0.030 (3)	0.086 (5)	0.076 (5)	0.007 (3)	0.011 (3)	−0.026 (4)
C112	0.033 (3)	0.061 (4)	0.063 (4)	0.000 (3)	0.004 (3)	−0.012 (3)
C201	0.022 (3)	0.046 (3)	0.024 (3)	−0.004 (2)	0.001 (2)	−0.003 (2)
C202	0.036 (3)	0.044 (3)	0.037 (3)	0.002 (3)	−0.006 (3)	−0.002 (3)
C203	0.054 (4)	0.057 (4)	0.046 (4)	0.001 (3)	−0.008 (3)	−0.019 (3)
C204	0.054 (4)	0.075 (5)	0.028 (3)	0.000 (3)	−0.001 (3)	−0.015 (3)
C205	0.049 (4)	0.071 (5)	0.032 (3)	−0.005 (3)	−0.005 (3)	0.008 (3)
C206	0.037 (3)	0.046 (3)	0.029 (3)	−0.001 (3)	−0.001 (2)	0.005 (2)
C207	0.036 (3)	0.035 (3)	0.025 (3)	0.004 (2)	0.005 (2)	0.005 (2)
C208	0.044 (3)	0.037 (3)	0.045 (3)	0.000 (3)	−0.010 (3)	0.002 (3)
C209	0.081 (5)	0.043 (4)	0.057 (4)	−0.001 (3)	−0.006 (4)	−0.008 (3)
C210	0.089 (6)	0.044 (4)	0.075 (5)	0.031 (4)	0.017 (5)	0.000 (4)
C211	0.053 (4)	0.063 (5)	0.076 (5)	0.020 (4)	0.010 (4)	0.009 (4)
C212	0.033 (3)	0.048 (4)	0.048 (4)	0.007 (3)	0.003 (3)	0.003 (3)
C301	0.026 (3)	0.043 (3)	0.027 (3)	−0.002 (2)	0.003 (2)	−0.005 (2)
C302	0.048 (4)	0.039 (3)	0.056 (4)	−0.004 (3)	0.000 (3)	−0.008 (3)
C303	0.069 (5)	0.045 (4)	0.078 (5)	0.000 (3)	−0.001 (4)	−0.012 (4)
C304	0.062 (5)	0.067 (5)	0.082 (6)	0.002 (4)	0.006 (4)	−0.042 (4)
C305	0.082 (5)	0.074 (5)	0.043 (4)	−0.020 (4)	0.022 (4)	−0.025 (4)
C306	0.068 (4)	0.051 (4)	0.036 (3)	−0.012 (3)	0.012 (3)	−0.010 (3)
C307	0.026 (3)	0.041 (3)	0.021 (3)	−0.004 (2)	0.004 (2)	−0.002 (2)
C308	0.030 (3)	0.049 (4)	0.055 (4)	−0.001 (3)	−0.001 (3)	0.016 (3)
C309	0.052 (4)	0.048 (4)	0.066 (4)	0.006 (3)	0.007 (3)	0.024 (3)
C310	0.057 (4)	0.057 (4)	0.045 (4)	−0.016 (3)	0.005 (3)	0.013 (3)
C311	0.034 (3)	0.067 (4)	0.048 (4)	−0.012 (3)	0.006 (3)	0.016 (3)
C312	0.037 (3)	0.048 (3)	0.043 (3)	0.000 (3)	0.006 (3)	0.010 (3)
C401	0.020 (2)	0.046 (3)	0.030 (3)	−0.003 (2)	0.000 (2)	0.005 (2)
C402	0.038 (3)	0.057 (4)	0.034 (3)	0.000 (3)	−0.008 (3)	0.003 (3)
C403	0.048 (4)	0.076 (5)	0.033 (3)	−0.010 (4)	−0.006 (3)	0.005 (3)
C404	0.042 (4)	0.077 (5)	0.044 (4)	−0.012 (3)	−0.019 (3)	0.021 (3)
C405	0.051 (4)	0.070 (5)	0.062 (5)	0.020 (3)	−0.019 (4)	0.010 (4)
C406	0.043 (4)	0.066 (4)	0.037 (3)	0.010 (3)	−0.001 (3)	0.000 (3)
C407	0.035 (3)	0.042 (3)	0.023 (3)	−0.005 (2)	−0.006 (2)	−0.001 (2)
C408	0.045 (3)	0.042 (3)	0.043 (3)	−0.002 (3)	−0.008 (3)	−0.002 (3)
C409	0.073 (5)	0.041 (4)	0.070 (5)	0.001 (3)	−0.014 (4)	0.001 (3)
C410	0.089 (6)	0.050 (4)	0.063 (5)	−0.018 (4)	0.016 (4)	0.002 (3)
C411	0.066 (5)	0.072 (5)	0.062 (5)	−0.033 (4)	0.025 (4)	−0.013 (4)
C412	0.053 (4)	0.049 (4)	0.043 (4)	−0.011 (3)	0.014 (3)	−0.010 (3)
C5	0.149 (11)	0.116 (8)	0.080 (7)	−0.014 (7)	0.015 (7)	0.006 (6)
Cl3	0.126 (2)	0.136 (2)	0.124 (2)	−0.0242 (19)	0.0329 (19)	−0.0336 (19)
Cl4	0.129 (3)	0.220 (5)	0.283 (6)	−0.001 (3)	−0.020 (4)	0.108 (4)
C6	0.127 (10)	0.090 (7)	0.156 (11)	−0.015 (7)	−0.019 (8)	−0.005 (7)
Cl5	0.161 (3)	0.121 (3)	0.184 (4)	−0.014 (2)	−0.012 (3)	−0.022 (2)
Cl6	0.201 (5)	0.178 (4)	0.233 (5)	−0.085 (3)	0.028 (4)	0.007 (3)
C7	0.224 (17)	0.070 (7)	0.219 (17)	−0.005 (9)	0.035 (14)	0.040 (9)
Cl7	0.274 (5)	0.103 (2)	0.104 (2)	0.013 (3)	0.029 (3)	0.0201 (17)
Cl8	0.132 (3)	0.147 (3)	0.268 (6)	−0.027 (2)	−0.003 (3)	−0.093 (3)

### (Bis{[(diphenylphosphanyl)methyl]diphenylphosphanylidene}methane-\ κ3P,C,P')carbonylchloridohydridoiridium(III) chloride dichloromethane trisolvate (complex1) Geometric parameters (Å, º)

**Table:**

Ir1—H1	1.54 (3)	C205—C206	1.393 (8)
Ir1—C4	1.891 (6)	C207—C212	1.387 (8)
Ir1—C1	2.157 (5)	C207—C208	1.401 (8)
Ir1—P4	2.3154 (13)	C208—C209	1.388 (9)
Ir1—P1	2.3438 (13)	C209—C210	1.375 (11)
Ir1—Cl1	2.4919 (14)	C210—C211	1.375 (11)
P1—C107	1.822 (5)	C211—C212	1.371 (9)
P1—C101	1.823 (5)	C301—C306	1.365 (8)
P1—C2	1.827 (5)	C301—C302	1.390 (8)
P2—C1	1.697 (5)	C302—C303	1.389 (9)
P2—C2	1.800 (5)	C303—C304	1.377 (11)
P2—C201	1.808 (5)	C304—C305	1.368 (11)
P2—C207	1.821 (5)	C305—C306	1.386 (9)
P3—C1	1.711 (5)	C307—C312	1.383 (7)
P3—C307	1.821 (5)	C307—C308	1.390 (8)
P3—C3	1.821 (5)	C308—C309	1.380 (8)
P3—C301	1.829 (5)	C309—C310	1.365 (9)
P4—C407	1.804 (5)	C310—C311	1.361 (9)
P4—C3	1.807 (5)	C311—C312	1.372 (8)
P4—C401	1.811 (5)	C401—C406	1.378 (8)
O1—C4	1.117 (7)	C401—C402	1.409 (8)
C101—C102	1.381 (8)	C402—C403	1.375 (8)
C101—C106	1.383 (8)	C403—C404	1.371 (10)
C102—C103	1.386 (9)	C404—C405	1.370 (10)
C103—C104	1.382 (10)	C405—C406	1.398 (8)
C104—C105	1.366 (10)	C407—C408	1.388 (8)
C105—C106	1.380 (9)	C407—C412	1.391 (8)
C107—C108	1.382 (8)	C408—C409	1.383 (9)
C107—C112	1.385 (8)	C409—C410	1.364 (11)
C108—C109	1.376 (9)	C410—C411	1.369 (11)
C109—C110	1.367 (9)	C411—C412	1.388 (9)
C110—C111	1.379 (10)	C5—Cl3	1.705 (11)
C111—C112	1.381 (9)	C5—Cl4	1.714 (12)
C201—C206	1.391 (7)	C6—Cl6	1.728 (12)
C201—C202	1.392 (7)	C6—Cl5	1.750 (13)
C202—C203	1.373 (8)	C7—Cl7	1.602 (15)
C203—C204	1.373 (9)	C7—Cl8	1.727 (17)
C204—C205	1.367 (9)		
			
H1—Ir1—C4	87.7 (18)	C109—C108—C107	121.6 (6)
H1—Ir1—C1	88.6 (18)	C110—C109—C108	120.2 (6)
C4—Ir1—C1	176.0 (2)	C109—C110—C111	119.4 (6)
H1—Ir1—P4	85.9 (19)	C110—C111—C112	120.3 (6)
C4—Ir1—P4	95.39 (16)	C111—C112—C107	120.9 (6)
C1—Ir1—P4	82.71 (14)	C206—C201—C202	118.8 (5)
H1—Ir1—P1	92.4 (19)	C206—C201—P2	123.2 (4)
C4—Ir1—P1	93.70 (16)	C202—C201—P2	118.0 (4)
C1—Ir1—P1	88.09 (14)	C203—C202—C201	119.9 (5)
P4—Ir1—P1	170.69 (5)	C204—C203—C202	121.3 (6)
H1—Ir1—Cl1	174 (2)	C205—C204—C203	119.6 (5)
C4—Ir1—Cl1	94.05 (19)	C204—C205—C206	120.2 (5)
C1—Ir1—Cl1	89.43 (14)	C201—C206—C205	120.2 (5)
P4—Ir1—Cl1	88.63 (5)	C212—C207—C208	119.7 (5)
P1—Ir1—Cl1	92.77 (5)	C212—C207—P2	120.6 (4)
C107—P1—C101	104.9 (2)	C208—C207—P2	119.5 (4)
C107—P1—C2	107.1 (2)	C209—C208—C207	119.3 (6)
C101—P1—C2	103.1 (2)	C210—C209—C208	119.9 (6)
C107—P1—Ir1	115.57 (18)	C209—C210—C211	120.6 (6)
C101—P1—Ir1	118.94 (18)	C212—C211—C210	120.3 (7)
C2—P1—Ir1	106.05 (16)	C211—C212—C207	120.1 (6)
C1—P2—C2	107.5 (2)	C306—C301—C302	120.0 (5)
C1—P2—C201	114.4 (2)	C306—C301—P3	122.1 (4)
C2—P2—C201	106.5 (2)	C302—C301—P3	117.8 (4)
C1—P2—C207	117.3 (3)	C303—C302—C301	119.3 (6)
C2—P2—C207	103.2 (2)	C304—C303—C302	119.9 (6)
C201—P2—C207	106.8 (2)	C305—C304—C303	120.6 (6)
C1—P3—C307	119.3 (2)	C304—C305—C306	119.6 (7)
C1—P3—C3	104.7 (2)	C301—C306—C305	120.6 (6)
C307—P3—C3	106.5 (2)	C312—C307—C308	118.3 (5)
C1—P3—C301	117.5 (2)	C312—C307—P3	121.3 (4)
C307—P3—C301	103.6 (2)	C308—C307—P3	120.4 (4)
C3—P3—C301	103.8 (2)	C309—C308—C307	120.9 (5)
C407—P4—C3	105.6 (2)	C310—C309—C308	119.5 (6)
C407—P4—C401	105.8 (2)	C311—C310—C309	120.2 (6)
C3—P4—C401	106.1 (2)	C310—C311—C312	121.0 (6)
C407—P4—Ir1	117.58 (18)	C311—C312—C307	120.0 (5)
C3—P4—Ir1	104.47 (17)	C406—C401—C402	118.2 (5)
C401—P4—Ir1	116.23 (17)	C406—C401—P4	123.1 (4)
P2—C1—P3	125.7 (3)	C402—C401—P4	118.6 (4)
P2—C1—Ir1	113.9 (3)	C403—C402—C401	121.0 (6)
P3—C1—Ir1	120.4 (3)	C404—C403—C402	119.8 (6)
P2—C2—P1	107.8 (3)	C405—C404—C403	120.6 (6)
P4—C3—P3	106.5 (3)	C404—C405—C406	120.1 (6)
O1—C4—Ir1	177.1 (6)	C401—C406—C405	120.4 (6)
C102—C101—C106	118.7 (5)	C408—C407—C412	118.3 (5)
C102—C101—P1	121.1 (4)	C408—C407—P4	122.2 (4)
C106—C101—P1	120.1 (5)	C412—C407—P4	119.4 (4)
C101—C102—C103	120.7 (6)	C409—C408—C407	121.1 (6)
C104—C103—C102	119.5 (6)	C410—C409—C408	119.8 (7)
C105—C104—C103	120.2 (6)	C409—C410—C411	120.2 (6)
C104—C105—C106	120.1 (6)	C410—C411—C412	120.6 (7)
C105—C106—C101	120.8 (6)	C411—C412—C407	119.8 (6)
C108—C107—C112	117.6 (5)	Cl3—C5—Cl4	114.8 (7)
C108—C107—P1	122.1 (4)	Cl6—C6—Cl5	111.8 (6)
C112—C107—P1	120.3 (4)	Cl7—C7—Cl8	116.3 (7)

### (Bis{[(diphenylphosphanyl)methyl]diphenylphosphanylidene}methane-\ κ3P,C,P')carbonylchloridohydridoiridium(III) chloride dichloromethane trisolvate (complex1) Hydrogen-bond geometry (Å, º)

**Table:**

*D*—H···*A*	*D*—H	H···*A*	*D*···*A*	*D*—H···*A*
C2—H2*B*···Cl2	0.98	2.60	3.534 (5)	160
C3—H3*B*···Cl2^i^	0.98	2.62	3.544 (5)	157
C5—H5*B*···Cl2^ii^	0.98	2.59	3.541 (10)	165
C6—H6*B*···Cl2^iii^	0.98	2.47	3.436 (10)	169
C7—H7*A*···Cl1^iv^	0.98	2.50	3.447 (15)	163
C105—H105···Cl1^v^	0.94	2.72	3.573 (8)	151
C312—H312···Cl2^i^	0.94	2.78	3.690 (6)	163

Symmetry codes: (i) *x*+1, *y*, *z*; (ii) *x*+1/2, −*y*+1/2, *z*+1/2; (iii) −*x*−1/2, *y*+1/2, −*z*+3/2; (iv) −*x*+1/2, *y*+1/2, −*z*+3/2; (v) −*x*, −*y*, −*z*+2.

### (Bis{[(diphenylphosphanyl)methyl]diphenylphosphanylidene}methane(1+)-κ3P,C,P')carbonylchloridohydridoiridium(III) dichloride–hydrochloric acid–water (1/2/5.5) (complex2) Crystal data

**Table:**

[IrClH(C_51_H_44_P_4_)(CO)]Cl_2_·2HCl·5.5H_2_O	*F*(000) = 5160
*M**_r_* = 1281.32	*D*_x_ = 1.518 Mg m^−^^3^
Monoclinic, *P*2_1_/*n*	Mo *K*α radiation, λ = 0.71073 Å
*a* = 19.7138 (2) Å	Cell parameters from 114911 reflections
*b* = 22.7327 (2) Å	θ = 1.0–26.0°
*c* = 25.3120 (3) Å	µ = 2.78 mm^−^^1^
β = 98.781 (1)°	*T* = 233 K
*V* = 11210.6 (2) Å^3^	Prism, colorless
*Z* = 8	0.3 × 0.2 × 0.06 mm

### (Bis{[(diphenylphosphanyl)methyl]diphenylphosphanylidene}methane(1+)-κ3P,C,P')carbonylchloridohydridoiridium(III) dichloride–hydrochloric acid–water (1/2/5.5) (complex2) Data collection

**Table:**

Nonius KappaCCD diffractometer	17290 reflections with *I* > 2σ(*I*)
Radiation source: fine-focus sealed tube	*R*_int_ = 0.037
Graphite monochromator	θ_max_ = 26.0°, θ_min_ = 1.2°
phi– and ω–scans	*h* = −24→22
68943 measured reflections	*k* = −28→28
22078 independent reflections	*l* = −26→31

### (Bis{[(diphenylphosphanyl)methyl]diphenylphosphanylidene}methane(1+)-κ3P,C,P')carbonylchloridohydridoiridium(III) dichloride–hydrochloric acid–water (1/2/5.5) (complex2) Refinement

**Table:**

Refinement on *F*^2^	Primary atom site location: structure-invariant direct methods
Least-squares matrix: full	Secondary atom site location: difference Fourier map
*R*[*F*^2^ > 2σ(*F*^2^)] = 0.033	Hydrogen site location: inferred from neighbouring sites
*wR*(*F*^2^) = 0.088	H-atom parameters constrained
*S* = 1.04	*w* = 1/[σ^2^(*F*_o_^2^) + (0.0372*P*)^2^ + 17.5174*P*] where *P* = (*F*_o_^2^ + 2*F*_c_^2^)/3
22078 reflections	(Δ/σ)_max_ = 0.004
1268 parameters	Δρ_max_ = 2.03 e Å^−^^3^
2 restraints	Δρ_min_ = −0.99 e Å^−^^3^

### (Bis{[(diphenylphosphanyl)methyl]diphenylphosphanylidene}methane(1+)-κ3P,C,P')carbonylchloridohydridoiridium(III) dichloride–hydrochloric acid–water (1/2/5.5) (complex2) Special details

**Table:**

Experimental. All data sets were measured with several scans to increase the number of redundant reflections. In our experience this method of averaging redundant reflections replaces in a good approximation semi-empirical absorptions methods (absorption correction programs like SORTAV lead to no better data sets).
Geometry. All esds (except the esd in the dihedral angle between two l.s. planes) are estimated using the full covariance matrix. The cell esds are taken into account individually in the estimation of esds in distances, angles and torsion angles; correlations between esds in cell parameters are only used when they are defined by crystal symmetry. An approximate (isotropic) treatment of cell esds is used for estimating esds involving l.s. planes.
Refinement. Two molecules in the asymmetric unit. Hydrogen atoms at Ir1 and Ir2 were found and refined isotropically. Hydrogens at C1 and C5 were also found but refined with bond restraints (d=0.98 angs.). Between the molecules is a network of hydrogen bonded water and hydrochloric acid molecules and chloride anions. The hydrogen atoms of these molecules could only partially found and were omitted. One water molecule (O4 and O4A) and one Hydrochloric acid or chloride (Cl10 and Cl1A) have a 1:1 position disorder. There are 8 Cl-positions in the network represented 4 chloride and 4 acid units.

### (Bis{[(diphenylphosphanyl)methyl]diphenylphosphanylidene}methane(1+)-κ3P,C,P')carbonylchloridohydridoiridium(III) dichloride–hydrochloric acid–water (1/2/5.5) (complex2) Fractional atomic coordinates and isotropic or equivalent isotropic displacement parameters (Å^2^)

**Table:**

	*x*	*y*	*z*	*U*_iso_*/*U*_eq_	Occ. (<1)
Ir1	0.181754 (6)	0.912702 (6)	0.415090 (5)	0.02126 (5)	
H11	0.2558 (18)	0.9271 (16)	0.4099 (14)	0.029 (10)*	
Ir2	0.321407 (6)	0.410832 (6)	0.593150 (5)	0.01959 (4)	
H22	0.2500 (17)	0.4236 (15)	0.6005 (14)	0.024 (9)*	
P1	0.21221 (5)	0.91856 (4)	0.50825 (4)	0.0253 (2)	
P2	0.29369 (4)	0.82093 (4)	0.47570 (4)	0.02331 (19)	
P3	0.21933 (4)	0.78277 (4)	0.36170 (3)	0.02198 (18)	
P4	0.16639 (5)	0.90008 (4)	0.32262 (4)	0.02340 (19)	
P5	0.28901 (5)	0.41950 (4)	0.50063 (3)	0.02245 (19)	
P6	0.20630 (4)	0.32196 (4)	0.53195 (3)	0.02165 (18)	
P7	0.27874 (4)	0.28207 (4)	0.64560 (3)	0.02201 (18)	
P8	0.33514 (4)	0.39775 (4)	0.68539 (3)	0.02260 (19)	
Cl1	0.06789 (4)	0.87577 (4)	0.42596 (4)	0.0324 (2)	
Cl2	0.43463 (4)	0.37404 (4)	0.58048 (4)	0.0325 (2)	
O1	0.13685 (16)	1.03896 (13)	0.40152 (13)	0.0493 (8)	
O2	0.37103 (15)	0.53572 (12)	0.60789 (12)	0.0477 (8)	
C1	0.22006 (17)	0.82147 (15)	0.42386 (13)	0.0227 (7)	
H1	0.1851 (14)	0.8033 (15)	0.4408 (13)	0.029 (10)*	
C2	0.25277 (19)	0.84808 (16)	0.52990 (14)	0.0297 (8)	
H2A	0.2868	0.8537	0.5620	0.036*	
H2B	0.2182	0.8200	0.5382	0.036*	
C3	0.21881 (17)	0.83659 (15)	0.30985 (13)	0.0240 (7)	
H3A	0.2003	0.8190	0.2753	0.029*	
H3B	0.2659	0.8495	0.3082	0.029*	
C4	0.15263 (19)	0.99113 (18)	0.40750 (15)	0.0329 (9)	
C5	0.27997 (16)	0.32091 (14)	0.58383 (13)	0.0199 (7)	
H5	0.3150 (13)	0.3018 (12)	0.5667 (11)	0.010 (8)*	
C6	0.24699 (18)	0.34970 (16)	0.47778 (14)	0.0268 (8)	
H6A	0.2127	0.3563	0.4460	0.032*	
H6B	0.2810	0.3214	0.4688	0.032*	
C7	0.27997 (17)	0.33607 (16)	0.69767 (13)	0.0261 (8)	
H7A	0.2972	0.3182	0.7323	0.031*	
H7B	0.2333	0.3503	0.6987	0.031*	
C8	0.35343 (19)	0.48842 (17)	0.60151 (14)	0.0296 (8)	
C101	0.27332 (19)	0.97435 (17)	0.53609 (16)	0.0337 (9)	
C102	0.2939 (2)	1.01953 (19)	0.50583 (18)	0.0449 (11)	
H102	0.2790	1.0207	0.4688	0.054*	
C103	0.3370 (3)	1.0636 (2)	0.5305 (2)	0.0622 (14)	
H103	0.3511	1.0945	0.5100	0.075*	
C104	0.3587 (3)	1.0616 (3)	0.5845 (2)	0.0651 (15)	
H104	0.3867	1.0919	0.6009	0.078*	
C105	0.3402 (3)	1.0163 (3)	0.6149 (2)	0.0635 (15)	
H105	0.3567	1.0150	0.6518	0.076*	
C106	0.2969 (2)	0.9722 (2)	0.59107 (17)	0.0491 (11)	
H106	0.2836	0.9412	0.6118	0.059*	
C107	0.14144 (19)	0.92914 (19)	0.54575 (15)	0.0351 (9)	
C108	0.1101 (2)	0.9837 (2)	0.54191 (19)	0.0544 (13)	
H108	0.1257	1.0132	0.5207	0.065*	
C109	0.0554 (3)	0.9949 (3)	0.5694 (2)	0.0755 (19)	
H109	0.0335	1.0317	0.5667	0.091*	
C110	0.0343 (3)	0.9514 (4)	0.6005 (2)	0.080 (2)	
H110	−0.0020	0.9590	0.6198	0.096*	
C111	0.0642 (3)	0.8977 (3)	0.6042 (2)	0.0721 (17)	
H111	0.0484	0.8684	0.6255	0.087*	
C112	0.1181 (2)	0.8857 (2)	0.57654 (18)	0.0501 (12)	
H112	0.1386	0.8483	0.5787	0.060*	
C201	0.33327 (18)	0.75168 (15)	0.49610 (14)	0.0281 (8)	
C202	0.40045 (19)	0.74022 (17)	0.48793 (15)	0.0321 (8)	
H202	0.4243	0.7678	0.4701	0.039*	
C203	0.4317 (2)	0.68814 (18)	0.50623 (18)	0.0415 (10)	
H203	0.4769	0.6802	0.5008	0.050*	
C204	0.3967 (2)	0.6481 (2)	0.5323 (2)	0.0554 (13)	
H204	0.4184	0.6129	0.5451	0.066*	
C205	0.3306 (3)	0.6586 (2)	0.5399 (2)	0.0663 (16)	
H205	0.3072	0.6307	0.5577	0.080*	
C206	0.2977 (2)	0.71047 (19)	0.5213 (2)	0.0485 (12)	
H206	0.2519	0.7174	0.5258	0.058*	
C207	0.36260 (17)	0.86855 (15)	0.46483 (14)	0.0265 (8)	
C208	0.37411 (18)	0.88632 (17)	0.41486 (15)	0.0302 (8)	
H208	0.3439	0.8747	0.3842	0.036*	
C209	0.4301 (2)	0.9212 (2)	0.40997 (19)	0.0450 (11)	
H209	0.4376	0.9339	0.3760	0.054*	
C210	0.4749 (2)	0.9374 (2)	0.4548 (2)	0.0542 (12)	
H210	0.5127	0.9615	0.4512	0.065*	
C211	0.4651 (2)	0.9188 (2)	0.5043 (2)	0.0537 (13)	
H211	0.4969	0.9291	0.5344	0.064*	
C212	0.4087 (2)	0.88487 (19)	0.51046 (16)	0.0401 (10)	
H212	0.4013	0.8729	0.5446	0.048*	
C301	0.28957 (18)	0.73269 (16)	0.36121 (14)	0.0275 (8)	
C302	0.28391 (19)	0.67840 (16)	0.38643 (16)	0.0340 (9)	
H302	0.2448	0.6698	0.4021	0.041*	
C303	0.3362 (2)	0.63748 (18)	0.38809 (18)	0.0435 (11)	
H303	0.3330	0.6010	0.4051	0.052*	
C304	0.3928 (2)	0.6506 (2)	0.3647 (2)	0.0519 (13)	
H304	0.4284	0.6228	0.3660	0.062*	
C305	0.3982 (2)	0.7031 (2)	0.3398 (2)	0.0522 (12)	
H305	0.4372	0.7109	0.3238	0.063*	
C306	0.3467 (2)	0.74543 (18)	0.33777 (17)	0.0399 (10)	
H306	0.3507	0.7818	0.3208	0.048*	
C307	0.14376 (18)	0.73816 (15)	0.34728 (14)	0.0278 (8)	
C308	0.09406 (19)	0.73260 (17)	0.38036 (17)	0.0355 (9)	
H308	0.0971	0.7545	0.4121	0.043*	
C309	0.0400 (2)	0.6943 (2)	0.3658 (2)	0.0501 (12)	
H309	0.0064	0.6900	0.3883	0.060*	
C310	0.0342 (2)	0.6626 (2)	0.3197 (2)	0.0577 (14)	
H310	−0.0034	0.6372	0.3103	0.069*	
C311	0.0834 (3)	0.6676 (2)	0.28666 (19)	0.0550 (13)	
H311	0.0793	0.6458	0.2548	0.066*	
C312	0.1387 (2)	0.70459 (19)	0.30041 (17)	0.0424 (10)	
H312	0.1729	0.7073	0.2784	0.051*	
C401	0.19720 (18)	0.95858 (15)	0.28336 (14)	0.0271 (8)	
C402	0.2406 (2)	1.00271 (19)	0.30682 (16)	0.0407 (10)	
H402	0.2550	1.0029	0.3440	0.049*	
C403	0.2623 (2)	1.0465 (2)	0.27505 (19)	0.0490 (11)	
H403	0.2909	1.0768	0.2910	0.059*	
C404	0.2426 (2)	1.0462 (2)	0.22070 (18)	0.0481 (11)	
H404	0.2575	1.0761	0.1995	0.058*	
C405	0.2011 (2)	1.0020 (2)	0.19749 (17)	0.0486 (11)	
H405	0.1877	1.0015	0.1602	0.058*	
C406	0.1787 (2)	0.95811 (19)	0.22858 (16)	0.0385 (9)	
H406	0.1506	0.9278	0.2122	0.046*	
C407	0.07960 (18)	0.88841 (16)	0.28820 (14)	0.0279 (8)	
C408	0.0603 (2)	0.84135 (19)	0.25456 (16)	0.0393 (10)	
H408	0.0919	0.8112	0.2509	0.047*	
C409	−0.0058 (2)	0.8386 (2)	0.22621 (18)	0.0516 (12)	
H409	−0.0189	0.8065	0.2034	0.062*	
C410	−0.0521 (2)	0.8828 (3)	0.23145 (19)	0.0555 (13)	
H410	−0.0967	0.8810	0.2120	0.067*	
C411	−0.0335 (2)	0.9290 (2)	0.2647 (2)	0.0555 (13)	
H411	−0.0653	0.9590	0.2682	0.067*	
C412	0.0319 (2)	0.9322 (2)	0.29335 (17)	0.0422 (10)	
H412	0.0442	0.9642	0.3164	0.051*	
C501	0.35689 (18)	0.43210 (16)	0.46057 (14)	0.0269 (8)	
C502	0.3884 (2)	0.48677 (19)	0.46588 (17)	0.0428 (10)	
H502	0.3748	0.5146	0.4897	0.051*	
C503	0.4395 (2)	0.5004 (2)	0.4363 (2)	0.0528 (13)	
H503	0.4614	0.5372	0.4403	0.063*	
C504	0.4581 (2)	0.4597 (3)	0.4011 (2)	0.0563 (14)	
H504	0.4920	0.4693	0.3801	0.068*	
C505	0.4279 (3)	0.4055 (2)	0.3963 (2)	0.0537 (13)	
H505	0.4417	0.3777	0.3725	0.064*	
C506	0.3774 (2)	0.39124 (19)	0.42598 (17)	0.0397 (10)	
H506	0.3569	0.3538	0.4227	0.048*	
C508	0.2069 (2)	0.51871 (19)	0.51128 (18)	0.0450 (11)	
H508	0.2200	0.5152	0.5484	0.054*	
C507	0.22857 (18)	0.47771 (16)	0.47726 (15)	0.0292 (8)	
C509	0.1657 (3)	0.5653 (2)	0.4902 (2)	0.0627 (14)	
H509	0.1515	0.5935	0.5133	0.075*	
C510	0.1457 (2)	0.5704 (2)	0.4363 (3)	0.0641 (15)	
H510	0.1190	0.6027	0.4225	0.077*	
C511	0.1645 (3)	0.5285 (3)	0.4022 (2)	0.0615 (14)	
H511	0.1489	0.5311	0.3653	0.074*	
C512	0.2067 (2)	0.4826 (2)	0.42257 (17)	0.0469 (11)	
H512	0.2206	0.4544	0.3992	0.056*	
C601	0.16664 (17)	0.25250 (15)	0.51261 (13)	0.0254 (8)	
C602	0.20494 (19)	0.20844 (17)	0.49293 (17)	0.0369 (9)	
H602	0.2517	0.2144	0.4910	0.044*	
C603	0.1733 (2)	0.15567 (18)	0.4762 (2)	0.0479 (11)	
H603	0.1988	0.1256	0.4629	0.057*	
C604	0.1046 (2)	0.14712 (18)	0.47905 (18)	0.0447 (11)	
H604	0.0833	0.1116	0.4669	0.054*	
C605	0.06694 (19)	0.19022 (17)	0.49950 (16)	0.0364 (9)	
H605	0.0205	0.1837	0.5023	0.044*	
C606	0.09772 (18)	0.24333 (16)	0.51602 (15)	0.0306 (8)	
H606	0.0720	0.2731	0.5295	0.037*	
C607	0.13787 (17)	0.37044 (15)	0.54243 (14)	0.0259 (8)	
C608	0.12795 (18)	0.38994 (17)	0.59230 (15)	0.0319 (8)	
H608	0.1584	0.3784	0.6228	0.038*	
C609	0.0731 (2)	0.42652 (19)	0.59750 (18)	0.0423 (10)	
H609	0.0669	0.4406	0.6314	0.051*	
C610	0.0277 (2)	0.4421 (2)	0.5530 (2)	0.0494 (12)	
H610	−0.0090	0.4675	0.5564	0.059*	
C611	0.0354 (2)	0.4211 (2)	0.5038 (2)	0.0498 (12)	
H611	0.0029	0.4306	0.4738	0.060*	
C612	0.09104 (19)	0.38598 (17)	0.49785 (16)	0.0352 (9)	
H612	0.0972	0.3726	0.4638	0.042*	
C701	0.20732 (18)	0.23359 (16)	0.64540 (15)	0.0295 (8)	
C702	0.1496 (2)	0.24920 (19)	0.66759 (17)	0.0411 (10)	
H702	0.1469	0.2859	0.6843	0.049*	
C703	0.0956 (2)	0.2087 (3)	0.6643 (2)	0.0605 (15)	
H703	0.0562	0.2179	0.6794	0.073*	
C704	0.0997 (3)	0.1558 (2)	0.6392 (2)	0.0637 (16)	
H704	0.0627	0.1295	0.6370	0.076*	
C705	0.1562 (3)	0.1405 (2)	0.61746 (19)	0.0538 (13)	
H705	0.1582	0.1039	0.6004	0.065*	
C706	0.2108 (2)	0.17939 (17)	0.62077 (17)	0.0388 (10)	
H706	0.2503	0.1690	0.6063	0.047*	
C707	0.35348 (18)	0.23694 (15)	0.66079 (14)	0.0277 (8)	
C708	0.40532 (19)	0.23352 (17)	0.62909 (16)	0.0346 (9)	
H708	0.4032	0.2560	0.5977	0.042*	
C709	0.4600 (2)	0.1961 (2)	0.6451 (2)	0.0511 (12)	
H709	0.4950	0.1927	0.6239	0.061*	
C710	0.4641 (2)	0.1638 (2)	0.6913 (2)	0.0597 (14)	
H710	0.5022	0.1392	0.7016	0.072*	
C711	0.4130 (3)	0.1673 (2)	0.7224 (2)	0.0560 (13)	
H711	0.4161	0.1453	0.7542	0.067*	
C712	0.3572 (2)	0.20293 (18)	0.70703 (17)	0.0413 (10)	
H712	0.3215	0.2044	0.7277	0.050*	
C801	0.30467 (17)	0.45851 (15)	0.72259 (14)	0.0263 (8)	
C802	0.32467 (19)	0.46072 (18)	0.77730 (15)	0.0338 (9)	
H802	0.3536	0.4314	0.7945	0.041*	
C803	0.3023 (2)	0.5058 (2)	0.80689 (17)	0.0467 (11)	
H803	0.3163	0.5073	0.8441	0.056*	
C804	0.2597 (2)	0.5484 (2)	0.78201 (19)	0.0481 (11)	
H804	0.2450	0.5794	0.8021	0.058*	
C805	0.2384 (2)	0.5459 (2)	0.72784 (18)	0.0490 (11)	
H805	0.2086	0.5748	0.7110	0.059*	
C806	0.2606 (2)	0.50079 (18)	0.69793 (16)	0.0389 (10)	
H806	0.2457	0.4990	0.6609	0.047*	
C807	0.42090 (18)	0.38327 (17)	0.72028 (14)	0.0295 (8)	
C808	0.4713 (2)	0.4244 (2)	0.71341 (17)	0.0417 (10)	
H808	0.4605	0.4561	0.6899	0.050*	
C809	0.5374 (2)	0.4186 (2)	0.7412 (2)	0.0557 (13)	
H809	0.5709	0.4471	0.7375	0.067*	
C810	0.5535 (2)	0.3709 (3)	0.7742 (2)	0.0593 (14)	
H810	0.5984	0.3665	0.7926	0.071*	
C811	0.5045 (3)	0.3298 (2)	0.78038 (19)	0.0580 (13)	
H811	0.5160	0.2973	0.8029	0.070*	
C812	0.4382 (2)	0.33597 (19)	0.75370 (17)	0.0436 (10)	
H812	0.4048	0.3077	0.7584	0.052*	
Cl3	0.43500 (8)	0.07777 (6)	0.35337 (6)	0.0683 (4)	
Cl4	0.63453 (6)	0.10085 (5)	0.24013 (5)	0.0502 (3)	
Cl5	0.36569 (5)	−0.10478 (5)	0.26820 (5)	0.0481 (3)	
Cl6	0.56146 (9)	−0.09005 (6)	0.14042 (6)	0.0753 (4)	
Cl7	0.61135 (7)	−0.23188 (6)	0.02459 (6)	0.0647 (3)	
Cl8	0.18768 (7)	−0.22306 (7)	0.18243 (5)	0.0737 (4)	
Cl9	0.11836 (6)	−0.26763 (6)	0.01405 (5)	0.0558 (3)	
Cl10	0.7388 (2)	0.2809 (2)	0.30806 (17)	0.1024 (16)	0.50
Cl1A	0.7741 (3)	0.2425 (3)	0.32103 (18)	0.130 (2)	0.50
O3	0.5264 (2)	0.18247 (18)	0.3666 (2)	0.0951 (14)	
O4	0.6467 (4)	0.1744 (4)	0.3440 (3)	0.075 (2)	0.50
O4A	0.6003 (4)	0.2120 (4)	0.2999 (4)	0.090 (3)	0.50
O5	0.82515 (19)	0.34084 (18)	0.39015 (14)	0.0707 (10)	
O6	0.43632 (19)	0.00727 (16)	0.25519 (15)	0.0694 (10)	
O7	0.5547 (2)	−0.01186 (16)	0.23911 (15)	0.0727 (11)	
O8	0.3074 (2)	−0.1457 (2)	0.15756 (16)	0.0925 (13)	
O9	0.3671 (2)	−0.2327 (2)	0.12315 (18)	0.0993 (14)	
O10	0.4977 (2)	−0.2185 (2)	0.11123 (19)	0.1075 (16)	
O11	0.2902 (2)	−0.31156 (18)	0.15257 (17)	0.0886 (13)	
O12	0.18821 (18)	−0.36714 (17)	0.09137 (15)	0.0699 (10)	
O13	0.05236 (17)	−0.24396 (15)	0.10971 (13)	0.0611 (9)	

### (Bis{[(diphenylphosphanyl)methyl]diphenylphosphanylidene}methane(1+)-κ3P,C,P')carbonylchloridohydridoiridium(III) dichloride–hydrochloric acid–water (1/2/5.5) (complex2) Atomic displacement parameters (Å^2^)

**Table:**

	*U*^11^	*U*^22^	*U*^33^	*U*^12^	*U*^13^	*U*^23^
Ir1	0.02149 (8)	0.01844 (8)	0.02403 (8)	0.00296 (5)	0.00408 (5)	−0.00136 (5)
Ir2	0.01927 (8)	0.01723 (8)	0.02234 (7)	−0.00200 (5)	0.00338 (5)	0.00099 (5)
P1	0.0255 (5)	0.0254 (5)	0.0253 (5)	0.0020 (4)	0.0047 (4)	−0.0042 (4)
P2	0.0222 (5)	0.0207 (5)	0.0265 (5)	0.0016 (4)	0.0021 (3)	0.0008 (4)
P3	0.0200 (4)	0.0187 (4)	0.0274 (5)	0.0008 (3)	0.0042 (3)	−0.0017 (3)
P4	0.0226 (5)	0.0231 (5)	0.0246 (5)	0.0017 (4)	0.0041 (3)	0.0009 (4)
P5	0.0244 (5)	0.0204 (5)	0.0231 (4)	−0.0021 (4)	0.0052 (3)	0.0025 (3)
P6	0.0210 (4)	0.0180 (4)	0.0257 (4)	−0.0011 (3)	0.0025 (3)	0.0000 (3)
P7	0.0211 (4)	0.0183 (4)	0.0267 (5)	0.0006 (3)	0.0039 (3)	0.0038 (3)
P8	0.0221 (5)	0.0221 (5)	0.0233 (4)	0.0003 (4)	0.0026 (3)	−0.0001 (4)
Cl1	0.0236 (4)	0.0342 (5)	0.0405 (5)	−0.0006 (4)	0.0082 (4)	−0.0009 (4)
Cl2	0.0225 (4)	0.0331 (5)	0.0431 (5)	0.0019 (4)	0.0088 (4)	0.0024 (4)
O1	0.0575 (19)	0.0251 (16)	0.063 (2)	0.0115 (14)	0.0014 (15)	−0.0042 (14)
O2	0.0563 (19)	0.0231 (16)	0.062 (2)	−0.0135 (14)	0.0038 (15)	0.0009 (14)
C1	0.0216 (18)	0.0210 (18)	0.0255 (18)	0.0034 (14)	0.0040 (13)	0.0010 (14)
C2	0.033 (2)	0.026 (2)	0.0299 (19)	0.0035 (16)	0.0035 (15)	0.0023 (15)
C3	0.0248 (18)	0.0233 (18)	0.0241 (18)	0.0004 (15)	0.0044 (13)	0.0008 (14)
C4	0.030 (2)	0.034 (2)	0.034 (2)	0.0032 (17)	0.0036 (16)	−0.0034 (17)
C5	0.0177 (17)	0.0152 (17)	0.0269 (18)	−0.0011 (13)	0.0044 (13)	0.0009 (13)
C6	0.031 (2)	0.0250 (19)	0.0246 (18)	−0.0032 (15)	0.0044 (14)	−0.0008 (14)
C7	0.0265 (19)	0.0275 (19)	0.0243 (18)	0.0016 (15)	0.0041 (14)	0.0022 (15)
C8	0.031 (2)	0.030 (2)	0.0268 (19)	0.0002 (16)	0.0007 (15)	0.0055 (15)
C101	0.028 (2)	0.032 (2)	0.040 (2)	−0.0001 (17)	0.0051 (16)	−0.0117 (17)
C102	0.041 (2)	0.040 (3)	0.050 (3)	−0.009 (2)	−0.0041 (19)	0.001 (2)
C103	0.053 (3)	0.048 (3)	0.083 (4)	−0.017 (2)	0.000 (3)	0.003 (3)
C104	0.046 (3)	0.065 (4)	0.082 (4)	−0.020 (3)	0.001 (3)	−0.031 (3)
C105	0.053 (3)	0.084 (4)	0.051 (3)	−0.020 (3)	0.001 (2)	−0.029 (3)
C106	0.048 (3)	0.061 (3)	0.038 (2)	−0.014 (2)	0.0077 (19)	−0.012 (2)
C107	0.031 (2)	0.048 (3)	0.0262 (19)	0.0023 (18)	0.0045 (15)	−0.0124 (18)
C108	0.053 (3)	0.061 (3)	0.051 (3)	0.022 (2)	0.009 (2)	−0.014 (2)
C109	0.050 (3)	0.104 (5)	0.072 (4)	0.030 (3)	0.005 (3)	−0.042 (4)
C110	0.035 (3)	0.144 (7)	0.063 (4)	0.001 (4)	0.012 (2)	−0.055 (4)
C111	0.052 (3)	0.115 (5)	0.056 (3)	−0.025 (3)	0.029 (3)	−0.025 (3)
C112	0.046 (3)	0.062 (3)	0.046 (3)	−0.008 (2)	0.020 (2)	−0.010 (2)
C201	0.030 (2)	0.0218 (19)	0.0320 (19)	0.0026 (15)	0.0018 (15)	0.0021 (15)
C202	0.031 (2)	0.028 (2)	0.037 (2)	−0.0013 (16)	0.0032 (16)	−0.0023 (16)
C203	0.032 (2)	0.032 (2)	0.058 (3)	0.0126 (18)	−0.0019 (18)	0.000 (2)
C204	0.050 (3)	0.035 (3)	0.079 (4)	0.016 (2)	0.006 (2)	0.020 (2)
C205	0.053 (3)	0.043 (3)	0.107 (5)	0.008 (2)	0.022 (3)	0.039 (3)
C206	0.035 (2)	0.033 (2)	0.080 (3)	0.0074 (19)	0.015 (2)	0.019 (2)
C207	0.0241 (18)	0.0218 (19)	0.033 (2)	0.0012 (15)	0.0032 (14)	−0.0011 (15)
C208	0.0237 (19)	0.030 (2)	0.037 (2)	−0.0011 (16)	0.0051 (15)	−0.0042 (16)
C209	0.037 (2)	0.046 (3)	0.057 (3)	−0.009 (2)	0.020 (2)	0.000 (2)
C210	0.039 (3)	0.049 (3)	0.075 (4)	−0.020 (2)	0.013 (2)	−0.010 (3)
C211	0.034 (2)	0.054 (3)	0.067 (3)	−0.014 (2)	−0.009 (2)	−0.014 (2)
C212	0.036 (2)	0.040 (2)	0.041 (2)	−0.0037 (19)	−0.0050 (17)	−0.0007 (19)
C301	0.0256 (19)	0.0235 (19)	0.0323 (19)	0.0042 (15)	0.0008 (14)	−0.0092 (15)
C302	0.034 (2)	0.025 (2)	0.042 (2)	0.0051 (16)	0.0012 (17)	−0.0049 (17)
C303	0.045 (3)	0.028 (2)	0.053 (3)	0.0117 (19)	−0.006 (2)	−0.0074 (19)
C304	0.039 (3)	0.042 (3)	0.069 (3)	0.021 (2)	−0.008 (2)	−0.022 (2)
C305	0.031 (2)	0.057 (3)	0.073 (3)	0.005 (2)	0.020 (2)	−0.019 (3)
C306	0.030 (2)	0.036 (2)	0.056 (3)	0.0007 (18)	0.0117 (18)	−0.0124 (19)
C307	0.0251 (18)	0.0210 (18)	0.036 (2)	−0.0013 (15)	−0.0007 (15)	0.0029 (15)
C308	0.028 (2)	0.027 (2)	0.052 (2)	0.0013 (16)	0.0080 (17)	0.0008 (18)
C309	0.032 (2)	0.042 (3)	0.077 (3)	−0.012 (2)	0.011 (2)	−0.001 (2)
C310	0.034 (3)	0.041 (3)	0.092 (4)	−0.016 (2)	−0.010 (2)	0.006 (3)
C311	0.062 (3)	0.044 (3)	0.053 (3)	−0.013 (2)	−0.011 (2)	−0.007 (2)
C312	0.048 (3)	0.037 (2)	0.040 (2)	−0.011 (2)	0.0022 (19)	−0.0063 (19)
C401	0.0264 (19)	0.0223 (19)	0.034 (2)	0.0056 (15)	0.0077 (15)	0.0038 (15)
C402	0.044 (2)	0.039 (2)	0.039 (2)	−0.0071 (19)	0.0053 (18)	0.0050 (19)
C403	0.050 (3)	0.041 (3)	0.057 (3)	−0.015 (2)	0.009 (2)	0.007 (2)
C404	0.050 (3)	0.046 (3)	0.051 (3)	−0.003 (2)	0.014 (2)	0.021 (2)
C405	0.052 (3)	0.059 (3)	0.036 (2)	−0.003 (2)	0.008 (2)	0.019 (2)
C406	0.041 (2)	0.042 (2)	0.033 (2)	−0.0042 (19)	0.0064 (17)	0.0045 (18)
C407	0.0245 (19)	0.032 (2)	0.0279 (19)	0.0016 (16)	0.0047 (14)	0.0075 (16)
C408	0.030 (2)	0.046 (3)	0.042 (2)	−0.0022 (18)	0.0018 (17)	−0.0048 (19)
C409	0.040 (3)	0.062 (3)	0.049 (3)	−0.011 (2)	−0.004 (2)	−0.006 (2)
C410	0.026 (2)	0.086 (4)	0.051 (3)	−0.002 (2)	−0.0063 (19)	0.016 (3)
C411	0.032 (2)	0.068 (3)	0.067 (3)	0.018 (2)	0.010 (2)	0.013 (3)
C412	0.030 (2)	0.046 (3)	0.050 (3)	0.0087 (19)	0.0025 (18)	−0.001 (2)
C501	0.0252 (19)	0.030 (2)	0.0261 (18)	0.0020 (16)	0.0067 (14)	0.0079 (15)
C502	0.048 (3)	0.039 (2)	0.043 (2)	−0.014 (2)	0.0130 (19)	0.0024 (19)
C503	0.043 (3)	0.057 (3)	0.058 (3)	−0.017 (2)	0.008 (2)	0.022 (2)
C504	0.037 (3)	0.079 (4)	0.057 (3)	0.007 (3)	0.019 (2)	0.031 (3)
C505	0.053 (3)	0.059 (3)	0.056 (3)	0.013 (2)	0.030 (2)	0.009 (2)
C506	0.038 (2)	0.041 (2)	0.044 (2)	0.0016 (19)	0.0203 (18)	0.0047 (19)
C508	0.043 (2)	0.035 (2)	0.054 (3)	0.0078 (19)	−0.004 (2)	−0.008 (2)
C507	0.0265 (19)	0.0222 (19)	0.038 (2)	−0.0019 (15)	0.0021 (15)	0.0069 (16)
C509	0.054 (3)	0.041 (3)	0.089 (4)	0.022 (2)	−0.001 (3)	−0.010 (3)
C510	0.040 (3)	0.046 (3)	0.102 (5)	0.013 (2)	0.000 (3)	0.027 (3)
C511	0.049 (3)	0.071 (4)	0.062 (3)	0.010 (3)	−0.001 (2)	0.033 (3)
C512	0.045 (3)	0.056 (3)	0.038 (2)	0.011 (2)	0.0052 (19)	0.015 (2)
C601	0.0259 (19)	0.0231 (19)	0.0269 (18)	−0.0046 (15)	0.0030 (14)	−0.0013 (14)
C602	0.028 (2)	0.029 (2)	0.054 (3)	−0.0029 (17)	0.0083 (17)	−0.0089 (18)
C603	0.050 (3)	0.024 (2)	0.073 (3)	−0.0040 (19)	0.019 (2)	−0.015 (2)
C604	0.043 (3)	0.027 (2)	0.062 (3)	−0.0133 (19)	0.004 (2)	−0.008 (2)
C605	0.028 (2)	0.033 (2)	0.047 (2)	−0.0098 (17)	0.0019 (17)	−0.0022 (18)
C606	0.029 (2)	0.027 (2)	0.036 (2)	−0.0036 (16)	0.0051 (15)	0.0001 (16)
C607	0.0203 (18)	0.0218 (18)	0.035 (2)	−0.0017 (14)	0.0027 (14)	0.0020 (15)
C608	0.0246 (19)	0.031 (2)	0.040 (2)	−0.0013 (16)	0.0051 (16)	0.0049 (17)
C609	0.039 (2)	0.034 (2)	0.058 (3)	0.0068 (19)	0.020 (2)	0.000 (2)
C610	0.033 (2)	0.039 (3)	0.077 (3)	0.013 (2)	0.009 (2)	0.005 (2)
C611	0.033 (2)	0.041 (3)	0.070 (3)	0.009 (2)	−0.009 (2)	0.012 (2)
C612	0.030 (2)	0.032 (2)	0.040 (2)	0.0032 (17)	−0.0048 (16)	0.0008 (17)
C701	0.0243 (19)	0.027 (2)	0.036 (2)	−0.0053 (15)	0.0001 (15)	0.0120 (16)
C702	0.031 (2)	0.045 (3)	0.048 (2)	−0.0027 (19)	0.0084 (18)	0.018 (2)
C703	0.030 (2)	0.079 (4)	0.073 (4)	−0.004 (2)	0.011 (2)	0.038 (3)
C704	0.047 (3)	0.057 (3)	0.079 (4)	−0.029 (3)	−0.014 (3)	0.037 (3)
C705	0.060 (3)	0.036 (3)	0.057 (3)	−0.019 (2)	−0.018 (2)	0.019 (2)
C706	0.039 (2)	0.026 (2)	0.047 (2)	−0.0073 (17)	−0.0048 (18)	0.0105 (18)
C707	0.0265 (19)	0.0186 (18)	0.036 (2)	0.0024 (15)	−0.0016 (15)	−0.0016 (15)
C708	0.028 (2)	0.027 (2)	0.050 (2)	0.0024 (16)	0.0099 (17)	0.0015 (17)
C709	0.031 (2)	0.042 (3)	0.081 (4)	0.011 (2)	0.011 (2)	−0.001 (2)
C710	0.043 (3)	0.042 (3)	0.087 (4)	0.019 (2)	−0.014 (3)	0.003 (3)
C711	0.064 (3)	0.041 (3)	0.057 (3)	0.019 (2)	−0.006 (2)	0.015 (2)
C712	0.047 (2)	0.035 (2)	0.041 (2)	0.0095 (19)	0.0052 (19)	0.0108 (19)
C801	0.0258 (19)	0.0237 (19)	0.0302 (19)	−0.0053 (15)	0.0070 (14)	−0.0037 (15)
C802	0.035 (2)	0.036 (2)	0.029 (2)	0.0003 (17)	0.0029 (16)	−0.0040 (17)
C803	0.048 (3)	0.057 (3)	0.034 (2)	0.001 (2)	0.0061 (19)	−0.015 (2)
C804	0.054 (3)	0.040 (3)	0.053 (3)	0.002 (2)	0.015 (2)	−0.020 (2)
C805	0.059 (3)	0.039 (3)	0.050 (3)	0.019 (2)	0.009 (2)	0.000 (2)
C806	0.046 (2)	0.035 (2)	0.034 (2)	0.0104 (19)	0.0016 (18)	−0.0025 (18)
C807	0.0250 (19)	0.034 (2)	0.0289 (19)	0.0032 (16)	0.0017 (14)	−0.0080 (16)
C808	0.029 (2)	0.054 (3)	0.041 (2)	−0.0022 (19)	−0.0009 (17)	0.001 (2)
C809	0.030 (2)	0.077 (4)	0.060 (3)	−0.009 (2)	0.004 (2)	−0.011 (3)
C810	0.030 (2)	0.089 (4)	0.055 (3)	0.018 (3)	−0.006 (2)	−0.018 (3)
C811	0.052 (3)	0.064 (3)	0.052 (3)	0.024 (3)	−0.011 (2)	0.003 (2)
C812	0.042 (2)	0.040 (3)	0.045 (2)	0.008 (2)	−0.0066 (19)	0.003 (2)
Cl3	0.0781 (9)	0.0606 (8)	0.0659 (8)	−0.0066 (7)	0.0099 (7)	−0.0135 (7)
Cl4	0.0385 (6)	0.0501 (7)	0.0642 (7)	−0.0033 (5)	0.0146 (5)	−0.0015 (5)
Cl5	0.0346 (6)	0.0519 (7)	0.0587 (7)	−0.0043 (5)	0.0099 (5)	0.0016 (5)
Cl6	0.1048 (12)	0.0631 (9)	0.0547 (8)	0.0212 (8)	0.0020 (7)	−0.0079 (6)
Cl7	0.0642 (8)	0.0604 (8)	0.0692 (8)	−0.0040 (6)	0.0092 (6)	−0.0160 (7)
Cl8	0.0698 (9)	0.1014 (12)	0.0506 (7)	−0.0067 (8)	0.0117 (6)	−0.0281 (7)
Cl9	0.0499 (7)	0.0702 (8)	0.0488 (7)	0.0051 (6)	0.0124 (5)	0.0078 (6)
Cl10	0.107 (3)	0.121 (4)	0.075 (2)	−0.061 (3)	0.000 (2)	0.017 (2)
Cl1A	0.151 (5)	0.174 (5)	0.071 (3)	−0.081 (4)	0.038 (3)	−0.053 (3)
O3	0.086 (3)	0.064 (3)	0.142 (4)	−0.002 (2)	0.041 (3)	−0.014 (3)
O4	0.078 (5)	0.082 (6)	0.068 (5)	−0.012 (4)	0.017 (4)	−0.005 (4)
O4A	0.098 (6)	0.076 (6)	0.108 (7)	−0.011 (5)	0.052 (5)	−0.019 (5)
O5	0.072 (2)	0.082 (3)	0.059 (2)	−0.003 (2)	0.0104 (18)	0.0081 (19)
O6	0.083 (3)	0.052 (2)	0.077 (3)	−0.0134 (19)	0.025 (2)	−0.0074 (18)
O7	0.088 (3)	0.057 (2)	0.080 (3)	−0.015 (2)	0.034 (2)	−0.0096 (19)
O8	0.106 (3)	0.100 (3)	0.070 (3)	−0.010 (3)	0.010 (2)	−0.003 (2)
O9	0.087 (3)	0.122 (4)	0.089 (3)	−0.009 (3)	0.014 (2)	−0.020 (3)
O10	0.092 (3)	0.124 (4)	0.110 (4)	−0.035 (3)	0.027 (3)	−0.009 (3)
O11	0.102 (3)	0.076 (3)	0.084 (3)	0.004 (2)	0.003 (2)	0.012 (2)
O12	0.065 (2)	0.078 (3)	0.068 (2)	0.001 (2)	0.0131 (18)	0.002 (2)
O13	0.064 (2)	0.060 (2)	0.061 (2)	−0.0007 (18)	0.0155 (17)	−0.0009 (17)

### (Bis{[(diphenylphosphanyl)methyl]diphenylphosphanylidene}methane(1+)-κ3P,C,P')carbonylchloridohydridoiridium(III) dichloride–hydrochloric acid–water (1/2/5.5) (complex2) Geometric parameters (Å, º)

**Table:**

Ir1—H11	1.52 (4)	C303—C304	1.372 (7)
Ir1—C4	1.874 (4)	C304—C305	1.362 (7)
Ir1—C1	2.207 (3)	C305—C306	1.394 (6)
Ir1—P4	2.3316 (9)	C307—C308	1.388 (5)
Ir1—P1	2.3466 (9)	C307—C312	1.401 (5)
Ir1—Cl1	2.4511 (9)	C308—C309	1.383 (6)
Ir2—H22	1.48 (3)	C309—C310	1.363 (7)
Ir2—C8	1.874 (4)	C310—C311	1.379 (7)
Ir2—C5	2.201 (3)	C311—C312	1.378 (6)
Ir2—P8	2.3281 (9)	C401—C406	1.379 (5)
Ir2—P5	2.3398 (9)	C401—C402	1.391 (5)
Ir2—Cl2	2.4500 (9)	C402—C403	1.388 (6)
P1—C101	1.816 (4)	C403—C404	1.371 (6)
P1—C107	1.819 (4)	C404—C405	1.371 (6)
P1—C2	1.837 (4)	C405—C406	1.385 (6)
P2—C207	1.791 (4)	C407—C408	1.384 (5)
P2—C201	1.797 (4)	C407—C412	1.389 (5)
P2—C1	1.802 (3)	C408—C409	1.389 (6)
P2—C2	1.803 (4)	C409—C410	1.377 (7)
P3—C3	1.793 (3)	C410—C411	1.362 (7)
P3—C307	1.793 (4)	C411—C412	1.381 (6)
P3—C301	1.794 (3)	C501—C506	1.379 (5)
P3—C1	1.801 (3)	C501—C502	1.386 (5)
P4—C407	1.817 (4)	C502—C503	1.379 (6)
P4—C401	1.819 (4)	C503—C504	1.374 (7)
P4—C3	1.832 (3)	C504—C505	1.367 (7)
P5—C507	1.818 (4)	C505—C506	1.375 (6)
P5—C501	1.821 (4)	C508—C507	1.380 (6)
P5—C6	1.842 (4)	C508—C509	1.390 (6)
P6—C607	1.793 (4)	C507—C512	1.390 (5)
P6—C601	1.797 (3)	C509—C510	1.366 (8)
P6—C5	1.803 (3)	C510—C511	1.373 (8)
P6—C6	1.805 (4)	C511—C512	1.385 (6)
P7—C701	1.787 (4)	C601—C606	1.390 (5)
P7—C707	1.789 (3)	C601—C602	1.391 (5)
P7—C7	1.799 (4)	C602—C603	1.388 (5)
P7—C5	1.799 (3)	C603—C604	1.382 (6)
P8—C807	1.814 (4)	C604—C605	1.377 (6)
P8—C801	1.824 (4)	C605—C606	1.387 (5)
P8—C7	1.830 (4)	C607—C608	1.380 (5)
O1—C4	1.135 (5)	C607—C612	1.390 (5)
O2—C8	1.134 (4)	C608—C609	1.386 (5)
C1—H1	0.960 (18)	C609—C610	1.375 (6)
C5—H5	0.972 (17)	C610—C611	1.364 (7)
C101—C102	1.379 (6)	C611—C612	1.383 (6)
C101—C106	1.399 (6)	C701—C706	1.387 (6)
C102—C103	1.397 (6)	C701—C702	1.390 (6)
C103—C104	1.369 (8)	C702—C703	1.401 (6)
C104—C105	1.368 (8)	C703—C704	1.367 (8)
C105—C106	1.394 (6)	C704—C705	1.362 (8)
C107—C112	1.379 (6)	C705—C706	1.386 (6)
C107—C108	1.382 (6)	C707—C708	1.395 (5)
C108—C109	1.394 (7)	C707—C712	1.395 (5)
C109—C110	1.368 (9)	C708—C709	1.385 (6)
C110—C111	1.354 (9)	C709—C710	1.371 (7)
C111—C112	1.385 (7)	C710—C711	1.375 (7)
C201—C206	1.383 (5)	C711—C712	1.373 (6)
C201—C202	1.396 (5)	C801—C806	1.379 (5)
C202—C203	1.382 (5)	C801—C802	1.382 (5)
C203—C204	1.371 (6)	C802—C803	1.380 (6)
C204—C205	1.367 (7)	C803—C804	1.371 (6)
C205—C206	1.392 (6)	C804—C805	1.373 (6)
C207—C208	1.379 (5)	C805—C806	1.384 (6)
C207—C212	1.406 (5)	C807—C812	1.378 (6)
C208—C209	1.379 (5)	C807—C808	1.394 (6)
C209—C210	1.378 (7)	C808—C809	1.390 (6)
C210—C211	1.364 (7)	C809—C810	1.375 (7)
C211—C212	1.381 (6)	C810—C811	1.370 (7)
C301—C306	1.381 (5)	C811—C812	1.384 (6)
C301—C302	1.402 (5)	Cl10—Cl1A	1.132 (7)
C302—C303	1.384 (5)	O4—O4A	1.582 (12)
			
H11—Ir1—C4	94.0 (14)	C206—C201—C202	120.1 (3)
H11—Ir1—C1	83.8 (14)	C206—C201—P2	119.8 (3)
C4—Ir1—C1	177.82 (15)	C202—C201—P2	120.1 (3)
H11—Ir1—P4	85.4 (13)	C203—C202—C201	119.6 (4)
C4—Ir1—P4	91.34 (12)	C204—C203—C202	120.0 (4)
C1—Ir1—P4	88.58 (9)	C205—C204—C203	120.7 (4)
H11—Ir1—P1	88.4 (13)	C204—C205—C206	120.4 (4)
C4—Ir1—P1	94.38 (12)	C201—C206—C205	119.1 (4)
C1—Ir1—P1	85.48 (9)	C208—C207—C212	120.0 (3)
P4—Ir1—P1	171.84 (3)	C208—C207—P2	123.6 (3)
H11—Ir1—Cl1	172.2 (14)	C212—C207—P2	116.3 (3)
C4—Ir1—Cl1	93.78 (12)	C207—C208—C209	119.8 (4)
C1—Ir1—Cl1	88.40 (9)	C210—C209—C208	120.1 (4)
P4—Ir1—Cl1	95.15 (3)	C211—C210—C209	120.7 (4)
P1—Ir1—Cl1	90.28 (3)	C210—C211—C212	120.4 (4)
H22—Ir2—C8	96.4 (13)	C211—C212—C207	119.0 (4)
H22—Ir2—C5	81.4 (13)	C306—C301—C302	120.5 (3)
C8—Ir2—C5	177.84 (14)	C306—C301—P3	123.4 (3)
H22—Ir2—P8	82.3 (13)	C302—C301—P3	116.1 (3)
C8—Ir2—P8	91.23 (11)	C303—C302—C301	119.7 (4)
C5—Ir2—P8	88.47 (9)	C304—C303—C302	119.5 (4)
H22—Ir2—P5	89.4 (13)	C305—C304—C303	121.0 (4)
C8—Ir2—P5	93.98 (11)	C304—C305—C306	121.0 (4)
C5—Ir2—P5	86.03 (9)	C301—C306—C305	118.4 (4)
P8—Ir2—P5	170.65 (3)	C308—C307—C312	119.7 (3)
H22—Ir2—Cl2	171.3 (13)	C308—C307—P3	124.7 (3)
C8—Ir2—Cl2	92.19 (12)	C312—C307—P3	115.5 (3)
C5—Ir2—Cl2	89.97 (9)	C309—C308—C307	118.9 (4)
P8—Ir2—Cl2	96.83 (3)	C310—C309—C308	121.5 (4)
P5—Ir2—Cl2	90.73 (3)	C309—C310—C311	120.1 (4)
C101—P1—C107	102.79 (18)	C312—C311—C310	120.0 (4)
C101—P1—C2	105.11 (18)	C311—C312—C307	119.8 (4)
C107—P1—C2	107.05 (19)	C406—C401—C402	119.1 (3)
C101—P1—Ir1	118.84 (14)	C406—C401—P4	119.0 (3)
C107—P1—Ir1	115.65 (12)	C402—C401—P4	121.9 (3)
C2—P1—Ir1	106.46 (12)	C403—C402—C401	119.6 (4)
C207—P2—C201	105.50 (16)	C404—C403—C402	120.8 (4)
C207—P2—C1	115.43 (16)	C405—C404—C403	119.5 (4)
C201—P2—C1	118.77 (16)	C404—C405—C406	120.4 (4)
C207—P2—C2	110.36 (17)	C401—C406—C405	120.5 (4)
C201—P2—C2	107.84 (17)	C408—C407—C412	119.0 (4)
C1—P2—C2	98.49 (16)	C408—C407—P4	124.0 (3)
C3—P3—C307	108.97 (16)	C412—C407—P4	116.8 (3)
C3—P3—C301	110.25 (17)	C407—C408—C409	120.1 (4)
C307—P3—C301	104.94 (17)	C410—C409—C408	120.0 (4)
C3—P3—C1	107.74 (16)	C411—C410—C409	120.2 (4)
C307—P3—C1	110.47 (17)	C410—C411—C412	120.5 (4)
C301—P3—C1	114.38 (16)	C411—C412—C407	120.2 (4)
C407—P4—C401	102.31 (16)	C506—C501—C502	119.7 (3)
C407—P4—C3	108.39 (16)	C506—C501—P5	124.0 (3)
C401—P4—C3	103.60 (16)	C502—C501—P5	116.3 (3)
C407—P4—Ir1	117.89 (12)	C503—C502—C501	120.1 (4)
C401—P4—Ir1	117.09 (12)	C504—C503—C502	119.4 (4)
C3—P4—Ir1	106.48 (11)	C505—C504—C503	120.5 (4)
C507—P5—C501	101.84 (16)	C504—C505—C506	120.4 (4)
C507—P5—C6	106.49 (17)	C505—C506—C501	119.7 (4)
C501—P5—C6	107.11 (17)	C507—C508—C509	119.5 (4)
C507—P5—Ir2	116.88 (13)	C508—C507—C512	119.3 (4)
C501—P5—Ir2	117.33 (12)	C508—C507—P5	122.6 (3)
C6—P5—Ir2	106.42 (11)	C512—C507—P5	118.1 (3)
C607—P6—C601	105.94 (16)	C510—C509—C508	120.7 (5)
C607—P6—C5	116.17 (16)	C509—C510—C511	120.3 (4)
C601—P6—C5	117.09 (16)	C510—C511—C512	119.6 (5)
C607—P6—C6	109.24 (17)	C511—C512—C507	120.5 (5)
C601—P6—C6	109.00 (16)	C606—C601—C602	120.2 (3)
C5—P6—C6	98.97 (16)	C606—C601—P6	120.2 (3)
C701—P7—C707	105.64 (17)	C602—C601—P6	119.5 (3)
C701—P7—C7	110.21 (17)	C603—C602—C601	119.2 (3)
C707—P7—C7	108.39 (16)	C604—C603—C602	120.3 (4)
C701—P7—C5	114.54 (16)	C605—C604—C603	120.5 (4)
C707—P7—C5	110.39 (16)	C604—C605—C606	119.8 (4)
C7—P7—C5	107.55 (16)	C605—C606—C601	119.9 (3)
C807—P8—C801	104.00 (16)	C608—C607—C612	119.5 (3)
C807—P8—C7	108.19 (17)	C608—C607—P6	123.2 (3)
C801—P8—C7	103.97 (16)	C612—C607—P6	117.2 (3)
C807—P8—Ir2	117.94 (12)	C607—C608—C609	120.1 (4)
C801—P8—Ir2	115.07 (12)	C610—C609—C608	119.7 (4)
C7—P8—Ir2	106.68 (11)	C611—C610—C609	120.6 (4)
H1—C1—P3	106 (2)	C610—C611—C612	120.1 (4)
H1—C1—P2	103 (2)	C611—C612—C607	119.9 (4)
P3—C1—P2	122.08 (19)	C706—C701—C702	120.5 (4)
H1—C1—Ir1	101 (2)	C706—C701—P7	117.1 (3)
P3—C1—Ir1	114.48 (16)	C702—C701—P7	122.3 (3)
P2—C1—Ir1	107.75 (16)	C701—C702—C703	118.0 (4)
P2—C2—P1	107.25 (19)	C704—C703—C702	120.5 (5)
P3—C3—P4	110.41 (18)	C705—C704—C703	121.6 (4)
O1—C4—Ir1	177.2 (4)	C704—C705—C706	119.2 (5)
H5—C5—P7	105.8 (18)	C705—C706—C701	120.2 (4)
H5—C5—P6	103.3 (18)	C708—C707—C712	120.1 (3)
P7—C5—P6	121.55 (18)	C708—C707—P7	124.1 (3)
H5—C5—Ir2	100.6 (18)	C712—C707—P7	115.8 (3)
P7—C5—Ir2	114.63 (16)	C709—C708—C707	118.2 (4)
P6—C5—Ir2	108.07 (15)	C710—C709—C708	121.4 (4)
P6—C6—P5	107.06 (18)	C709—C710—C711	120.4 (4)
P7—C7—P8	109.90 (18)	C712—C711—C710	119.8 (4)
O2—C8—Ir2	177.3 (4)	C711—C712—C707	120.2 (4)
C102—C101—C106	119.8 (4)	C806—C801—C802	119.5 (3)
C102—C101—P1	122.6 (3)	C806—C801—P8	121.9 (3)
C106—C101—P1	117.5 (3)	C802—C801—P8	118.6 (3)
C101—C102—C103	119.8 (4)	C803—C802—C801	120.3 (4)
C104—C103—C102	119.8 (5)	C804—C803—C802	119.9 (4)
C105—C104—C103	121.2 (4)	C803—C804—C805	120.1 (4)
C104—C105—C106	119.6 (5)	C804—C805—C806	120.2 (4)
C105—C106—C101	119.7 (4)	C801—C806—C805	119.9 (4)
C112—C107—C108	119.8 (4)	C812—C807—C808	119.0 (4)
C112—C107—P1	123.3 (3)	C812—C807—P8	124.4 (3)
C108—C107—P1	116.9 (3)	C808—C807—P8	116.5 (3)
C107—C108—C109	120.0 (5)	C809—C808—C807	120.3 (4)
C110—C109—C108	118.8 (5)	C810—C809—C808	119.6 (4)
C111—C110—C109	121.6 (5)	C811—C810—C809	120.3 (4)
C110—C111—C112	120.1 (6)	C810—C811—C812	120.4 (5)
C107—C112—C111	119.6 (5)	C807—C812—C811	120.4 (4)

### (Bis{[(diphenylphosphanyl)methyl]diphenylphosphanylidene}methane(1+)-κ3P,C,P')carbonylchloridohydridoiridium(III) dichloride–hydrochloric acid–water (1/2/5.5) (complex2) Hydrogen-bond geometry (Å, º)

**Table:**

*D*—H···*A*	*D*—H	H···*A*	*D*···*A*	*D*—H···*A*
C1—H1···Cl1	0.96 (3)	2.82 (3)	3.252 (3)	109 (2)
C3—H3*A*···Cl8^i^	0.98	2.51	3.466 (4)	164
C3—H3*B*···Cl5^i^	0.98	2.57	3.493 (4)	158
C6—H6*A*···O8^ii^	0.98	2.59	3.431 (5)	144
C6—H6*B*···Cl9^ii^	0.98	2.82	3.746 (4)	158
C7—H7*A*···Cl1*A*^iii^	0.98	2.73	3.614 (6)	150
C7—H7*B*···Cl4^iii^	0.98	2.60	3.518 (4)	157
C206—H206···Cl7^iii^	0.94	2.79	3.719 (4)	172
C310—H310···Cl4^ii^	0.94	2.83	3.714 (4)	158
C602—H602···Cl9^ii^	0.94	2.62	3.557 (4)	179
C704—H704···Cl1^iv^	0.94	2.82	3.534 (6)	134
C708—H708···Cl2	0.94	2.80	3.503 (4)	132
C710—H710···Cl5^v^	0.94	2.72	3.614 (4)	160
C712—H712···Cl10^iii^	0.94	2.81	3.734 (6)	167

Symmetry codes: (i) *x*, *y*+1, *z*; (ii) −*x*+1/2, *y*+1/2, −*z*+1/2; (iii) *x*−1/2, −*y*+1/2, *z*+1/2; (iv) −*x*, −*y*+1, −*z*+1; (v) −*x*+1, −*y*, −*z*+1.
